# Theta oscillations regulate the speed of locomotion via a hippocampus to lateral septum pathway

**DOI:** 10.1038/ncomms9521

**Published:** 2015-10-12

**Authors:** Franziska Bender, Maria Gorbati, Marta Carus Cadavieco, Natalia Denisova, Xiaojie Gao, Constance Holman, Tatiana Korotkova, Alexey Ponomarenko

**Affiliations:** 1 Behavioural Neurodynamics Group, Leibniz-Institut für Molekulare Pharmakologie (FMP), Robert-Rössle-Street 10, Berlin 13125, Germany; 2 NeuroCure Cluster of Excellence, Charité Universitätsmedizin, Charitéplatz 1, Virchowweg 6, CCO, Berlin 10117, Germany

## Abstract

Hippocampal theta oscillations support encoding of an animal's position during spatial navigation, yet longstanding questions about their impact on locomotion remain unanswered. Combining optogenetic control of hippocampal theta oscillations with electrophysiological recordings in mice, we show that hippocampal theta oscillations regulate locomotion. In particular, we demonstrate that their regularity underlies more stable and slower running speeds during exploration. More regular theta oscillations are accompanied by more regular theta-rhythmic spiking output of pyramidal cells. Theta oscillations are coordinated between the hippocampus and its main subcortical output, the lateral septum (LS). Chemo- or optogenetic inhibition of this pathway reveals its necessity for the hippocampal regulation of running speed. Moreover, theta-rhythmic stimulation of LS projections to the lateral hypothalamus replicates the reduction of running speed induced by more regular hippocampal theta oscillations. These results suggest that changes in hippocampal theta synchronization are translated into rapid adjustment of running speed via the LS.

## Abstract (web summary)

Hippocampal theta oscillations support encoding of spatial information during navigation, yet their role in locomotion is poorly understood. Here the authors demonstrate that hippocampal theta oscillations regulate the speed of locomotion in rodents through a hippocampo-lateral septal-hypothalamic pathway.

## Graphical abstract

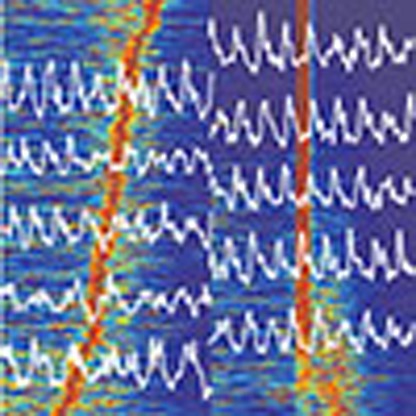

During locomotion, hippocampal theta oscillations (5–12 Hz) accompany spatial navigation^1^,^2^,^3^, yet there are longstanding questions open about the role of the hippocampus (Hip) and theta oscillations in locomotion^4^,^5^ via rhythmic coordination of specific brain circuits. Hippocampal theta rhythms require the integrity of the medial septum (MS) and can be modulated by subcortical inputs depending on ongoing behaviour^6^,^7^. Although theta oscillations accompany running and their frequency changes with running speed^8^,^9^,^10^,^11^, studies using lesions or electrical stimulation have revealed a complex relationship between theta synchronization and motor output, suggesting contradictory roles for the hippocampal theta rhythm in locomotion^5^,^12^,^13^.

In contrast to extensively characterized hippocampo-cortical interactions^1^,^3^, little is known about interactions of the Hip with its main subcortical output target, the lateral septum (LS)^14^. The LS is a key element in circuits governing expression of innate behaviours according to environmental context^15^,^16^. Lesions or inactivation of LS lead to hyperactivity^15^, and a major LS target, the lateral hypothalamus (LH), comprises the diencephalic locomotion region, which provides downstream motor circuits with direct command for movement^17^,^18^. However, the specific functions of the Hip–LS pathway as well as that of its downstream projections to LH remain elusive.

Here we manipulated hippocampal theta oscillations in freely behaving mice by optogenetic stimulation of MS GABAergic cells' axons in the Hip, while simultaneously estimating the fidelity of theta oscillations entrainment using electrophysiological recordings. We show that higher regularity of theta oscillations leads to running with a less variable and slower speed during exploratory behaviour. We further demonstrate theta-rhythmic coordination between Hip and LS. By combining optogenetic control of hippocampal theta oscillations with axonal chemogenetic (DREADDs) or optogenetic (eNpHR3.0) inhibition of Hip–LS pathway, as well as using optogenetic activation of LS–LH projections, we show the role of Hip–LS–LH pathway in theta-rhythmic regulation of locomotion.

## Results

### Optogenetic control of hippocampal theta oscillations

**Figure 1:**
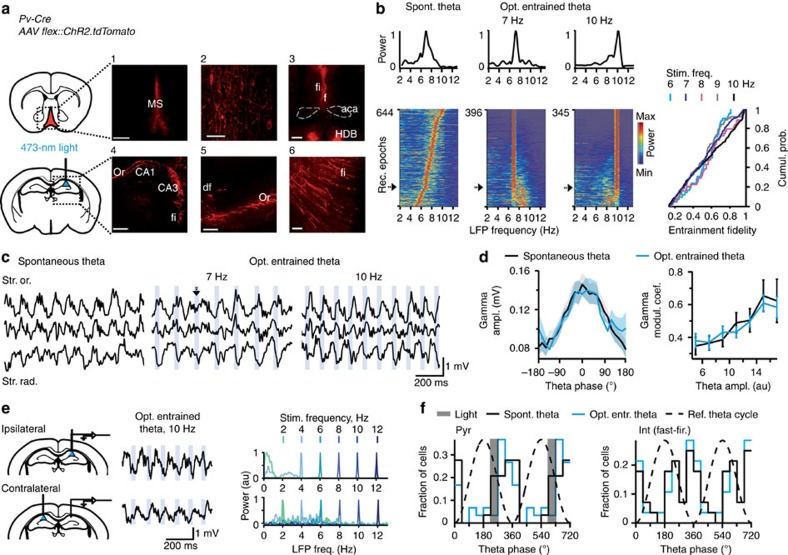
Optogenetic control of hippocampal theta oscillations. (**a**) Injections of Cre-dependent ChR2 in MS of *PV-Cre* mice and light-induced stimulation of MS–Hip projections. Expression of *AAV2/1.CAGGS.flex.ChR2.tdTomato.WPRESV40*: neuronal somata in MS (1,2), fibre tracts, fimbria (fi)-fornix (f), nucleus of the horizontal limb of the diagonal band (HDB) (3,5,6) and axons in the Hip (4,5). Scale bars, 500 μm (1,3,4) and 50 μm (2,5,6). (**b**) Left: LFP PSD (colour coded, computed for 10-s epochs) for all control recordings, as well as optostimulation at 7 and 10 Hz (*N*=9 mice). Power spectra marked with arrows are shown on top. The rows are ordered according to entrainment fidelity (optogenetically entrained theta), or dominant theta frequency (spontaneous theta). Right: cumulative distribution of theta entrainment fidelity for various optostimulation frequencies. (**c**) Laminar LFP profiles of spontaneous and optogenetically controlled theta oscillations; str. or., stratum oriens; str.rad., stratum radiatum. It is worth noting that the light pulse marked by an arrowhead (at 7 Hz) resets theta phase, thus adjusting the rhythm frequency to the stimulation frequency. (**d**) Phase–amplitude coupling of theta and gamma oscillations (left) compared across theta amplitudes (right) between spontaneous and optogenetically entrained theta (*P*=0.42, *N*=3 mice, 8 and 19 recordings, respectively). Data are presented as mean±s.e.m. (**e**) Intact bilateral coordination of optogenetically entrained theta oscillations (*P*<0.05, Pearson's correlation, *N*=5 mice). Left: signal traces recorded during ipsi- (top) and contralateral (bottom) hippocampal stimulation; right: respective LFP power spectra during optostimulation. (**f**) Left: histograms of preferred discharge phases of CA1 pyramidal cells (opt. entrainment: blue, 30 neurons; spontaneous theta: black, 29 neurons). Grey shaded bar: a theta phase bin when timestamps of laser pulse were preferentially recorded. Right: histograms of preferred discharge phases of fast firing interneurons (*n*=28 neurons). Preferred theta phases did not differ (pyramidal cells, *P*=0.79, Watson–Williams test; fast firing interneurons, *P*=0.97, Watson U2 permutation test).

To study the role of hippocampal theta oscillations in locomotion, we developed a preparation that enabled precise temporal control of hippocampal theta rhythm in freely behaving mice. GABAergic cells in the MS were selectively targeted by introducing a Cre-dependent ChR2 virus into the MS of parvalbumin (*PV*)*-Cre* mice (Fig. 1a and Supplementary Figs 1 and 2a,b). We selectively stimulated axons of MS GABAergic cells in the dorsal Hip via an optic fibre implanted above the CA1 area. Optogenetic stimulation of GABAergic septo-hippocampal projections at theta frequencies elicited theta oscillations (Fig. 1b,c). Simultaneous electrophysiological recordings in the Hip allowed us to compute the efficacy of optogenetic entrainment of theta oscillations with high temporal precision (Fig. 1b). Theta oscillations frequency matched theta-band frequencies of laser pulses (6–12 Hz) as indicated by high entrainment fidelity (>0.3), that is, the concentration of the local field potential (LFP) power around the stimulation frequency, in >80% of recordings (Fig. 1b and Supplementary Fig. 2c). In contrast, optostimulation at non-theta frequencies was less effective for entrainment of hippocampal oscillations (Supplementary Fig. 2c).

Optogenetically controlled theta oscillations possessed features of physiological theta oscillations in the mouse (for example, see ref. 19), including characteristic laminar phase profiles (Fig. 1c), phase-amplitude coupling with gamma oscillations (coefficient of modulation, *F*_4,8_=1.04, *P*=0.42; Fig. 1d, see also Supplementary Methods), bilateral coordination (contralateral entrainment, Pearson's *r*=0.99±0.0004, *P*<0.05 in each of five out of six mice; Fig. 1e), typical changes of power along septo-temporal axis (power, *F*_1,15_=0.25, *P*=0.62; Supplementary Fig. 2d), as well as unchanged preferential firing phases of recorded in the CA1 area putative pyramidal cells and interneurons (pyramidal cells: *F*_1,57_=0.08, *P*=0.79, Watson–Williams test (Fig. 1f and Supplementary Fig. 2e,f); fast-firing interneurons^20^: *P*=0.97, Watson U2 permutation test (Fig. 1f and Supplementary Fig. 2g,h, see also Supplementary Fig. 2i)). During spontaneous and optogenetically entrained theta oscillations, firing rates did not differ for pyramidal cells (*t*_10_=−0.017, *P*=0.98), fast-firing interneurons (*t*_27_=−0.045, *P*=0.96) and str. oriens-theta trough interneurons^21^,^22^ (*t*_9_=−0.19, *P*=0.85; Supplementary Fig. 2j). Positional firing properties of pyramidal cells (Supplementary Fig. 3a) were unaffected by optogenetic stimulation as shown by unchanged spatial coherence (*t*_71_=−0.40, *P*=0.69), sparsity (*t*_71_=0.83, *P*=0.41; Supplementary Fig. 3b) and by similar spatial firing of the same pyramidal cells during control light compared with optogenetic stimulation epochs (peak firing rates, *P*=0.10, Wilcoxon signed rank test; correlations of firing rates in the same spatial locations, *t*_30_=−0.67, *P*=0.51; Supplementary Fig. 3c–e).

### Hippocampal theta oscillations regulate running speed

**Figure 2:**
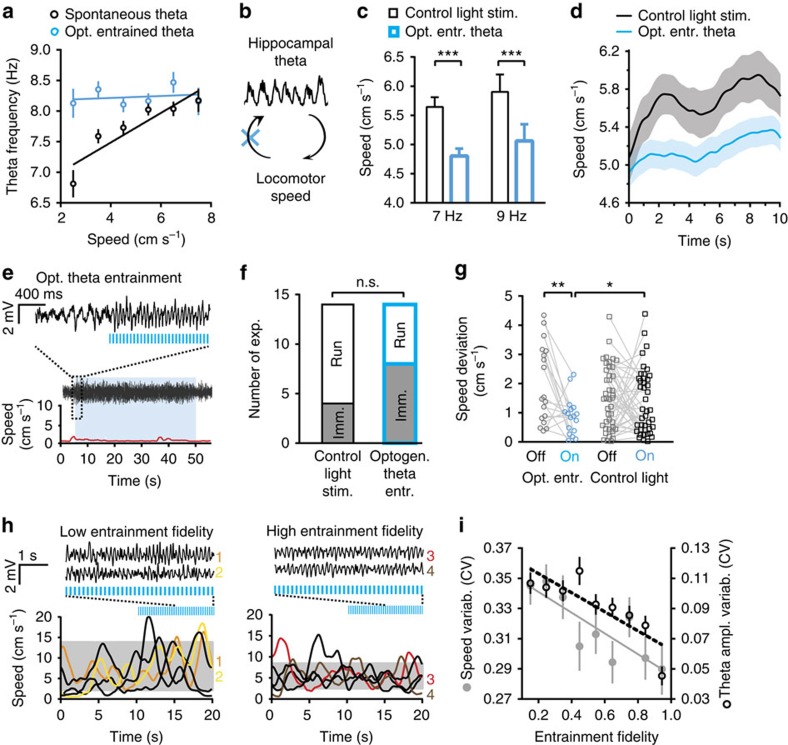
Optogenetic entrainment of hippocampal theta oscillations regulates locomotion speed and speed variability. (**a**) Correlation of running speed and frequency of theta oscillations during spontaneous (*r*=0.82, Pearson's correlation, *P*=0.013, *N*=6 mice, *n*=42 recording sessions) but not optogenetically entrained theta oscillations (*r*=0.04, *P*=0.7, *N*=8 mice, *n*=72 recording sessions). (**b**) Scheme illustrating a bidirectional influence between theta and locomotion as proposed earlier^4^,^8^,^10^. Optogenetic control of theta removes the influence of speed-correlated afferents on oscillation frequency (blue cross), as oscillation frequency is controlled by the laser pulse frequency. (**c**) Optogenetic entrainment of theta oscillations at 7 Hz (*P*=0.0002, Bonferroni test, *N*=5 mice) or 9 Hz (*P*=0.0002, *N*=3 mice) reduced speed in running mice. (**d**) Running speed during 10-s epochs of optogenetically entrained (blue) and spontaneous (black) theta oscillations. (**e**) Example of optogenetically elicited theta oscillations during immobility. Hippocampal LFP signal traces (2–250 Hz band-pass filtered, middle), recorded simultaneously with running speed measurement (red trace, bottom). Blue shade marks time of optostimulation. Excerpt, top: LFP signal trace shown at a higher time resolution. (**f**) Number of experiments when resting mice stayed immobile (Imm.) or moved (Run) during 15 s after stimulation onset (*P*=0.13, *χ*^2^-test, *N*=8 mice, optogenetic entrainment; *N*=5 mice, control light stimulation). (**g**) Speed (deviation from group mean) averaged for 20 s before and after the onset of optogenetic theta entrainment (left, *N*=8 mice) or control light stimulation (right, *N*=13 mice). Speed was less variable during optogenetic theta entrainment compared with baseline (*P*=0.0062, Bonferroni test, *n*=18 recording sessions, *N=8* mice) and control light stimulation recordings (*P*=0.0107, *n*=42 recording sessions, *N*=13 mice). (**h**) Representative traces before and after the onset of stimulation (7 Hz) with a low (<0.3; left) or high (>0.8; right) entrainment fidelity; grey shadows mark 10%–90% ranges of speed distributions during stimulation. (**i**) Higher entrainment fidelity was associated with lower coefficients of variation of theta amplitude (*r*=−0.84, *P*=0.0046) and running speed (*r*=−0.84, Pearson's correlation, *P*=0.0051, *n*=79 recording sessions, *N*=8 mice). *P<0.05, **P<0.01, ***P<0.001. Data are presented as mean±s.e.m.

Activity of speed-correlated hippocampal afferents was proposed to influence frequency of theta oscillations^8^,^9^,^10^. In agreement, in our baseline recordings theta frequency was correlated with running speed (Pearson's *r*=0.82, *P*=0.013; Fig. 2a). Gaining control over the oscillations frequency using optogenetically induced entrainment of theta oscillations eliminated the influence of speed-controlled afferents on oscillations frequency. Accordingly, correlation between theta frequency and running speed was absent during theta entrainment (Pearson's *r*=0.04, *P*=0.7), as the oscillations' frequency was determined by that of light pulses and therefore was not affected by the animal's speed (Fig. 2a,b). This allowed us to examine reverse causality, that is, whether hippocampal theta oscillations themselves can affect locomotion speed. Optogenetic entrainment of theta oscillations modified average speed of running mice. The speed was lower during running epochs, when the frequency of theta oscillations was optogenetically set to 7 or 9 Hz, than during control light stimulation epochs of running with spontaneous theta oscillations of the respective frequency (frequency-matched theta, *F*_1,248_=17.85, *P*=0.00003; 7 Hz: *P*=0.0002; 9 Hz: *P*=0.0002; Fig. 2c), as well as of more broad, 5–10 Hz, theta band frequencies (*F*_1,323_=18.88, *P*=0.00002; Supplementary Fig. 4a, see also Fig. 2d and Supplementary Fig. 4b). Control light stimulation was identical to the opsin-activating stimulation, except for that it was delivered through a dummy cable that did not allow the light to penetrate the surface of the brain^23^. The exposure to the same amount of light in the environment allowed to control for possible influence of visual stimulation on movement. No changes in time of exploration (*F*_1,244_=0.37, *P*=0.54) and duration of continuous running (*F*_1,244_=0.002, *P*=0.97) were found during optogenetically entrained theta at 7 and 9 Hz (Supplementary Fig. 4c). The reduction of running speed was specific for entrainment of theta oscillations as indicated by the lack of effect of stimulation outside the theta band, at 2, 4 or 20 Hz (*F*_2,84_=0.07, *P*=0.8; Supplementary Fig. 4d, see also Supplementary Methods) and of continuous 1 s pulses (*F*_1,15_=1.22, *P*=0.29; Supplementary Fig. 4e).

Next, as spontaneous hippocampal theta oscillations are associated with locomotor activity, we elicited theta oscillations during immobility to study whether induction of hippocampal theta oscillations can initiate movement. Optostimulation induced hippocampal theta oscillations (Fig. 2e and Supplementary Fig. 4f), whereas control light stimulation did not (Supplementary Fig. 4g). Nevertheless, optogenetically induced theta was not associated with higher likelihood of running initiation (*χ*^2^-test, *χ*^2^(1)=2.33, *P*=0.13; Fig. 2f). Therefore, optogenetic induction of hippocampal theta oscillations did not initiate locomotion in immobile mice.

To examine the immediate effect of the optogenetically controlled theta oscillations on locomotion, we measured running speed on optostimulation onset preceded by either running or immobility. Irrespective of baseline speed (*F*_58,58_=1.12, *P*=0.33, repeated-measures analysis of variance), entrainment of theta oscillations locked average running speed to a relatively narrow range, rendering it less variable than during control light stimulation (*F*_1,102_=6.59, *P*=0.027, Bonferroni test: *P*=0.0107; Fig. 2g and Supplementary Fig. 4h), or during the prestimulation baseline (Bonferroni test: *P*=0.0062). Speed variability did not differ between control light stimulation and spontaneous locomotion without light stimulation (*F*_41,41_=0.9, *P*=0.75; Supplementary Fig. 4i). Furthermore, speed variability was lower when theta was optogenetically entrained with high fidelity in comparison with low-fidelity entrainment (Pearson's *r*=−0.84, *P*=0.0051; Fig. 2h,i). Rhythmic optogenetic stimulation at non-theta frequencies (at 2, 4 and 20 Hz) did not change running speed variability (*F*_1,131_=0.43, *P*=0.51; Supplementary Fig. 4j), whereas 1 s continuous light pulses even increased it (*F*_1,15_=4.67, *P*=0.047; Supplementary Fig. 4k). These results indicated that optogenetically entrained theta rhythm stabilized locomotion and prompted identification of hippocampal theta oscillations' parameters, other than oscillations' frequency (7 versus 9 Hz: *F*_1,248_=0.82, *P*=0.37; Fig. 2c), which regularized running speed.

**Figure 3:**
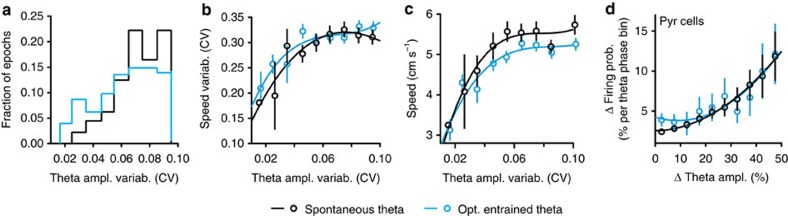
Variability of theta amplitude mediates speed variability. (**a**) Distribution of theta oscillation epochs according to variability of theta cycles amplitude (spontaneous theta, black, *n*=384 recording epochs, *N*=8 mice; optogenetic stimulation, blue, *n*=818 recording epochs, *N=8* mice). (**b**,**c**) Both in control and during optogenetic theta entrainment, theta amplitude variability predicted changes of speed variability (**b**, polynomial fit, *R*^2^=0.89, spontaneous theta; *R*^2^=0.90, optogenetic stimulation) and of running speed (**c**, *R*^2^=0.96, spontaneous theta; *R*^2^=0.92, optogenetic stimulation). (**d**) Changes of theta amplitude correlated with changes of firing probability in CA1 pyramidal cells during spontaneous and optogenetically entrained theta (25 and 12 single units, respectively; polynomial fit, *R*^2^=0.90, spontaneous theta; *R*^2^=0.88, optogenetic stimulation). Data are presented as mean±s.e.m.

In addition to setting the dominant frequency of theta oscillations, optogenetic entrainment controlled the temporal regularity of the theta rhythm, that is, variability (coefficient of variation (CV)) of frequency and amplitude. Optogenetically entraining theta with higher fidelity generated oscillations of more constant frequency (Fig. 1b) and amplitude (Pearson's correlation: *r*=−0.84, *P*=0.0046; Fig. 2i). Further, we examined whether increased regularity of spontaneous theta oscillations is also associated with more regular locomotion speed. We grouped epochs of spontaneous theta as well as theta during optogenetic stimulation according to theta amplitude variability (Fig. 3a) and averaged running speed and its variability within theta amplitude variability bins (Fig. 3b,c). Although the proportion of low-amplitude variability epochs was higher for optogenetically entrained theta, spontaneous and optogenetically entrained theta oscillations displayed a similar range of amplitude variability (Fig. 3a). Less variable theta amplitude was accompanied by a lower running speed variability, both during spontaneous and optogenetically entrained theta (Fig. 3b and Supplementary Table 1). Running speed also correlated with the amplitude variability of spontaneous theta oscillations (Fig. 3c and Supplementary Fig. 4l) and with optogenetically induced changes in theta amplitude variability (*P*=0.0001; Supplementary Table 1). In contrast to these findings in dorsal Hip, theta oscillations in a more ventral part of Hip (Supplementary Fig. 5a,b) did not display consistent association with locomotion (Supplementary Fig. 5c,d), in line with reported in rats differences along the septo-temporal axis^24^. All together, these results suggest that the variability of theta oscillation amplitude is a natural parameter, which regulates running speed and its variability during spontaneous theta oscillations as well.

To elucidate properties of hippocampal pyramidal cell output associated with more regular theta oscillations, we related changes in the number of spikes emitted near theta peaks or troughs (that is, comparing phase bins with similar excitability) to differences in the LFP amplitudes of respective cycles. Half of recorded pyramidal cells changed their firing probability depending on the amplitude difference between theta cycles (*P*<0.05 for 46% of 55 cells during spontaneous theta and 55% of 22 cells during optogenetically entrained theta; Fig. 3d, see also Supplementary Methods), indicating that theta oscillations with increased amplitude regularity were accompanied by a more temporally regular theta-rhythmic population output of pyramidal cells.

### Hip–LS–LH pathway mediates theta regulation of speed

**Figure 4:**
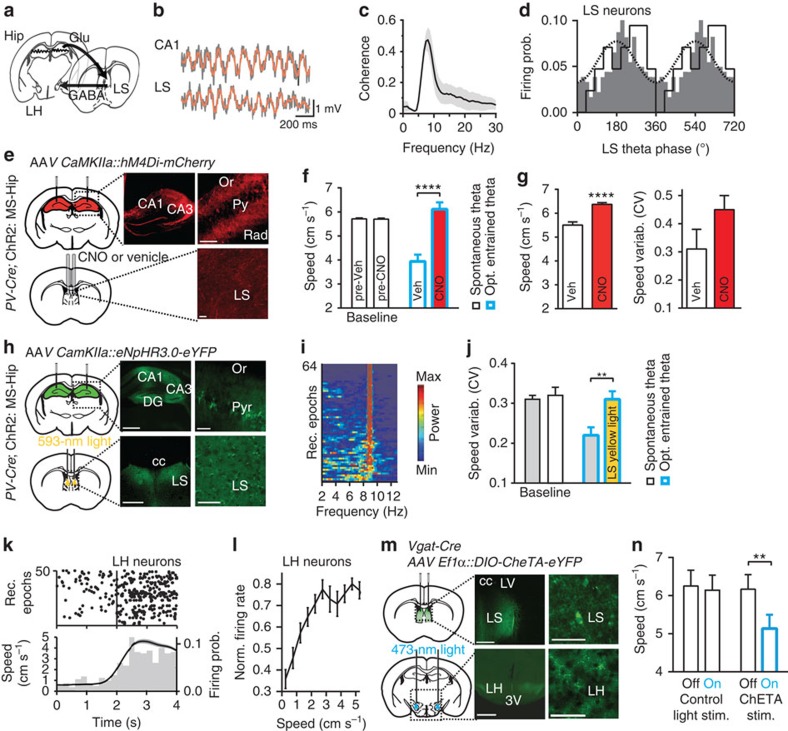
Hip–LS-LH pathway supports theta-rhythmic speed regulation. (**a**) Hip–LS–LH pathway. (**b**) Coordinated theta oscillations in Hip and LS (LFP, 1–200 Hz, grey trace; 5–15 Hz, brown trace, band-pass filtered). (**c**) Coherence of the Hip and LS LFP during hippocampal theta oscillations (*N*=3 mice). (**d**) Histograms of preferred LS theta discharge phases of 21 out of 73 significantly theta-modulated LS units (black line, *P*<0.05, Rayleigh test). Grey bars, firing probability of an example unit; dotted line, reference oscillation cycle. (**e**) Top: DREADDs (*AAV-CaMKIIa-hM4D(Gi)-mCherry*) expression in hippocampal pyramidal cells. Bottom: cannula implantations for CNO/vehicle injections and axonal immunofluorescence in LS. Scale bars, 500 μm (left) and 50 μm (right). (**f**) Intra-LS CNO, in comparison with intra-LS vehicle, prevented reduction of running speed during optogenetic theta entrainment (*P*<0.00001, analysis of variance (ANOVA)). Left bars show baseline running speed. (**g**) Running speed (*P*<0.00001, ANOVA; see also Supplementary Fig. 6f) and speed variability (*P*=0.15, *N*=6 mice) after intra-LS CNO or vehicle during spontaneous theta. (**h**) Top: eNpHR3.0 (*AAV2/1.CamKIIa.eNpHR3.0-EYFP.WPRE.hGH*) expression in hippocampal pyramidal cells. Bottom: bilateral optic fibres implantation, axonal immunofluorescence in LS. Scale bars, 500 μm (left) and 50 μm (right). (**i**) PSD (colour coded) for all recordings where optogenetic theta entrainment at 9 Hz was combined with eNpHR3.0 stimulation in LS. Rows are ordered according to entrainment fidelity. (**j**) Reduction of speed variability during optogenetic theta entrainment (grey, *P*=0.0073, ANOVA, *N*=8 mice) was prevented by simultaneous LS yellow light (593 nm) delivery (yellow bar, baseline white bar). (**k**,**l**) Locomotion-dependent firing of LH cells. (**k**) Top: examples of running onset—triggered rastergrams (50 epochs, 11 units, epochs are ordered according to firing rate); bottom: spike count (grey bars) and average running speed (black line). (**l**) Changes of firing rate according to running speed (*P*<0.05 for each cell, Pearson's correlation, 11 cells). (**m**) Top: injections and expression of Cre-dependent ChETA (*AAV2/5.Ef1a.DIO.ChETA(E123T/H134R)-EYFP.WPRE.hGH*) in LS in *Vgat-Cre* mice. Bottom: bilateral fibre implantation and axonal fluorescence in LH. Scale bars, 500 μm (left) and 50 μm (right). (**n**) Optogenetic theta-frequency activation of LS–LH pathway decreased running speed (*P*=0.003, Bonferroni test, *N*=7 mice). Data are presented as mean±s.e.m.

Hippocampal pyramidal cells send prominent projections to subcortical regions via the LS^14^ (Fig. 4a). We thus examined theta-rhythmic coordination of Hip and LS using dual-site LFP and LS unitary recordings (Fig. 4b–d and Supplementary Fig. 6a,b). LFP coherence between the hippocampal CA1 area and LS displayed the dominant maximum at theta frequencies (Fig. 4b,c). Approximately one-third of LS cells revealed theta-rhythmic activity—their firing was locked to LS theta oscillations during exploration (*P*<0.05 for 29% of 73 cells, Rayleigh test; Fig. 4d, see also Supplementary Methods).

To ascertain the contribution of the Hip to LS (Hip–LS) pathway in theta-mediated regulation of locomotion, we inhibited this pathway using chemo- or optogenetics. First, inhibitory DREADDs (hM4Di, designer receptors exclusively activated by clozapine-*N*-oxide, CNO), which transiently inactivate axonal terminals upon local CNO injection^25^ (Supplementary Fig. 6c), were bilaterally expressed in hippocampal pyramidal cells (Fig. 4e and Supplementary Figs 1 and 6d,e), whereas ChR2 was expressed in GABAergic MS cells as described above. In contrast to the observation of the reduced running speed during optogenetic theta entrainment when the Hip–LS pathway was intact (intra-LS vehicle injection), after intra-LS CNO injection running speed did not decrease during the theta rhythmic stimulation (*F*_1,18211_=514.94, *P*<0.00001; Fig. 4f). Furthermore, during exploration accompanied by spontaneous theta (without optogenetic stimulation), intra-LS injection of CNO resulted in an increased running speed (*F*_1,10809_=29.23, *P*<0.00001; Fig. 4g and Supplementary Fig. 6f) and in a nonsignificant increase of speed variability (*F*_1,13_=2.64, *P*=0.15; Fig. 4g).

To manipulate the Hip–LS pathway at a shorter time scale (<1 min) and thus address the origin of fast changes of locomotion, that is, running speed variability, an inhibitory opsin, halorhodopsin (eNpHR3.0), was bilaterally expressed in hippocampal pyramidal cells (Fig. 4h and Supplementary Fig. 1, 6g,h), whereas ChR2 was expressed in MS GABAergic cells. Yellow light (593 nm) was delivered bilaterally on hippocampal projections in LS to inhibit the Hip–LS pathway, whereas theta oscillations were optogenetically entrained, as in previous experiments (Figs 1, 2, 3), by the blue light in the Hip (Fig. 4i). When the Hip–LS pathway was inhibited, the entrainment of theta oscillations did not evoke speed variability reduction, as observed in mice with an intact Hip–LS pathway (*F*_1,36_=8.38, *P*=0.0073; Fig. 4j). Neither during control yellow light irradiation nor during LS yellow light application in NpHR3.0(−) mice was the running speed reduced when hippocampal theta oscillations were entrained (*F*_2,66_=0.037, *P*=0.96; Supplementary Fig. 6i). Thus, intact transmission in the Hip–LS pathway is required for the adjustment of running speed by theta oscillations.

The LS sends inhibitory projections to subcortical regions implicated in control of locomotion, including the LH^14^. We found that 65% of 17 recorded LH neurons displayed locomotion-dependent firing: they increased discharge during initiation of locomotion and maintained increased firing during spontaneous running (Pearson's correlation, firing rate versus running speed, *P*<0.05 for each of 11 neurons, Fig. 4k,l and Supplementary Fig. 6j,k). To study the effect of theta-rhythmic excitation of LS–LH projections on locomotion, an engineered excitatory opsin (ChETA)^26^ was selectively expressed in LS GABAergic cells in *Vgat-Cre* mice and blue light was delivered on projections of these cells in the LH (Fig. 4m and Supplementary Fig. 1). Optogenetic stimulation of LS projections to LH at theta frequency (9 Hz) led to a decrease of average speed (light × stimulation type interaction, *F*_1,32_=4.69, *P*=0.04; Bonferroni test: LH stimulation: *P*=0.003, control stimulation: *P*=0.99; Fig. 4n). Thus, optogenetic stimulation of projections from LS to LH decreased locomotion, suggesting that theta-rhythmic input from Hip via LS to LH regulates running speed.

## Discussion

By optogenetically controlling hippocampal theta oscillations in freely behaving mice, we found that higher regularity of theta oscillations' amplitude and frequency was associated with more stable discharge probabilities of pyramidal cells and regularized locomotion, leading to running with a less variable and slower speed. This pattern was also evident in spontaneous theta oscillations where higher regularity of theta amplitude was also accompanied by steadier and slower locomotion. We also demonstrated the coordination of theta oscillations in the Hip with its main subcortical output region, the LS. Finally, using projection-specific opto- and chemogenetic manipulations, we show the role of this pathway and projections of LS to the LH in theta-rhythmic regulation of locomotion.

Association between dorsal hippocampal theta rhythm and exploratory activity is one of the most extensively studied examples of coordination between brain rhythms and behaviour^4^,^5^,^9^,^10^,^27^,^28^,^29^,^30^,^31^. Their relationship is often referred as behavioural state dependent, implying the influence of afferents, bearing sensory and motor information, on circuits involved in theta rhythm generation^6^,^8^,^13^. These include hippocampal and cortical networks, which receive, amplify and/or generate theta rhythm, ascending neuromodulatory pathways, as well as subcortical theta rhythm generators^6^. Behavioural state-dependent activity of MS cells, interconnected with subcortical regions, including several hypothalamic nuclei^11^,^32^, is thought to underlie matching of theta frequency to changing running speed^33^. Hippocampal theta oscillations' amplitude is correlated with movement magnitude^4^,^34^ and with the speed of steady running but not with the speed of running evoked by stimulation of speed-correlated hippocampal inputs^13^. This suggests that coordination between changes of theta oscillations amplitude and locomotion may, at least in part, be brought about by other mechanisms than the activity of speed-related hippocampal afferents, which connects frequency of theta with running speed.

The modulation of hippocampal theta oscillations according to behavioural state does not rule out the possibility of the reverse influence, that is, of theta oscillations on the motor output^4^. Indeed, regulation of hippocampal theta oscillations by behaviour-dependent subcortical inputs^11^ and the presence of prominent direct descending projections of Hip^14^ naturally raises the question of whether the Hip provides functionally significant feedback to subcortical regions during theta state. An earlier study used electrical stimulation of MS to induce theta oscillatory patterns in the Hip independent of behaviour, thus eliminating confounding correlations, caused by motor activity^35^. They found dissociation of theta rhythm from its behavioural correlates, suggesting that theta oscillations do not directly induce locomotion. On the other hand, MS inactivation or activation resulted in reduced or increased locomotion, respectively^13^, whereas electrical stimulation of Hip led to the inhibition of voluntary movement^12^. Using cell type- and projection-specific control of hippocampal theta oscillations in combination with running speed estimation, our results demonstrate that theta-rhythmic signalling in the Hip does not change behavioural state from resting to spontaneous exploration in freely behaving mice, but regulates ongoing locomotion.

The optogenetic preparation implemented in our study enabled real-time control of the hippocampal theta rhythm by combining ChR2 expression in GABAergic MS cells that are crucial for theta rhythm generation^36^, optogenetic stimulation of their axons in the Hip and electrophysiological monitoring. In contrast to theta induction by the electrical stimulation of the MS^37^, theta entrainment using our protocol was accompanied not only by intact macroscopic features of the oscillation but also by unchanged firing rates, theta discharge phases and spatial firing properties of the hippocampal neurons. GABAergic cells in MS express PV and are believed to play a key role in the generation and maintenance of hippocampal theta rhythm^19^,^36^,^38^,^39^,^40^. PV cells in the MS provide extensive collateral innervation within the MS^39^ and their projections to the Hip selectively target the interneurons^41^. Theta-rhythmic inhibition of hippocampal basket interneurons by bursting MS PV cells^39^ is thought to determine periodic discharge of pyramidal cells^42^, which, via MS-projecting interneurons^43^,^44^,^45^,^46^,^47^, provide rhythmic feedback to MS and thus contributes to initiation and maintenance of the theta rhythm^36^. From this perspective, spontaneous and optogenetically controlled theta oscillations shared physiological mechanisms but differed in the precision of rhythmic timing signals, necessary for triggering and maintaining theta rhythm. Interplay of the optogenetically induced rhythm with intrinsic synchronization dynamics in the Hip probably leads to variability of optogenetic entrainment during stimulation of MS inputs. Interactions of optogenetically induced and intrinsic rhythmic signals, resulted in more regular oscillation epochs during high entrainment, and less regular ones otherwise. This experimentally evoked theta rhythm variability mimicked natural changes of theta regularity brought about by hippocampal processing of cortical and subcortical inputs and by interactions between intrahippocampal theta rhythm generators^1^,^6^. Quantification of the entrainment fidelity allowed for precise estimation of theta oscillations' control by optogenetic stimulation as opposed to modulation by behaviour-related inputs.

We propose that unravelling the impact of theta oscillations on locomotion requires: first, controlling theta oscillations, thus weakening or eliminating their dependence on internal, that is, behaviour-related, signals; second, revealing changes in locomotion measures according to degree of external oscillations' control; third, network oscillation features, crucial for locomotion regulation, should correlate with locomotion measures not only during externally controlled, but also during spontaneous theta oscillations. Addressing these points, we found, first, that running speed was not correlated with the frequency of optogenetically entrained theta oscillations, indicating that gaining control of the rhythm eliminated the influence of speed-correlated afferents. Second, during episodes when optogenetic stimulation was delivered at non-theta frequencies and did not entrain theta, speed was not affected; furthermore, changes in running speed were proportional to the degree of optogenetic theta entrainment (that is, low versus high fidelity; Fig. 2h,i). Third, theta amplitude regularity correlated with running speed and its regularity both during spontaneous and optogenetically entrained theta oscillations. The degree of optogenetically induced entrainment determined systematic changes of theta variability. Further, both optogenetically manipulated and spontaneous changes of the theta variability predicted running speed variability, indicating a physiologically relevant influence. Hence, regularity of theta oscillations adjusts running speed, while theta frequency and amplitude do not regulate locomotion, but are rather set by speed-related afferents.

Although changes of exploratory behaviour due to possible alterations of spatial representations by hippocampal networks during optogenetic entrainment cannot be entirely excluded, unchanged characteristics of pyramidal cells' spatial firing suggest that spatial navigation was not impaired. Further, perturbations that disrupt hippocampal function, such as lesions, are known to increase running speed^48^, in contrast to effects evoked by optogenetic theta entrainment. Hippocampal dysfunction is also associated with an unspecific increase of locomotor activity^49^, whereas the changes observed here were confined to reduced running speed and its variability, while other aspects of explorative behaviour remained unchanged.

Hippocampal theta frequency synchronization operates at time scales relevant for various brain functions. An animal's path is represented by place cells in accordance with the running speed^50^, within single theta cycles at a millisecond timescale of synaptic plasticity^30^, whereas rhythmic variations of excitability over tens of milliseconds assist coupling of rhythms^29^,^51^ and communication between brain regions^52^. We show that changes in amplitude of theta oscillations at a slower, from hundreds of milliseconds to seconds, scale regulate locomotion and are associated with modifications of the theta phase—matched firing probabilities of pyramidal cells. This suggests that population discharge probabilities display higher temporal stability during theta oscillations of increased regularity as opposed to epochs of theta with less regular amplitude. Differences in the firing rate of place cells arise from global remapping of representations upon changes of spatial environment, from rate remapping due to variability of sensory input^53^,^54^ and reward representations^55^,^56^, or from encoding of different types and sequences of memory episodes occurring in the same location^57^,^58^. As firing of pyramidal cell ensembles in the dorsal Hip contributes to spatiotemporal pattern of LFP theta oscillations^59^, the degree of theta oscillations' regularity may, at least in part, reflect experience-dependent flexibility of hippocampal representations. Our results show that hippocampal theta oscillations regulate ongoing locomotion but alone do not change the state of the animal from immobile to spontaneous exploration. However, depending on behavioural context, hippocampal theta oscillations, jointly with other brain regions involved in motivation, arousal and motor programme selection, may be necessary for initiation of, for instance, goal-directed locomotion^16^,^18^. Grastyan *et al*.^5^ and Vanderwolf^4^ implicated changes in theta rhythmic activity in motivational aspects of running and in behavioural control of complex voluntary movements including locomotion, respectively. Our findings provide the first evidence, obtained using function-selective manipulation of theta oscillations, reconciling these diverse perspectives.

Here we show that the dorsal Hip is coordinated with its main subcortical target, the LS, through theta oscillations. Coordination of the Hip with the LS relies on non-reciprocal convergent inputs from hippocampal pyramidal cells to LS GABAergic neurons^14^. Chemogenetic inhibition of Hip–LS projections in the present study resulted in the increased running speed, pointing to a circuitry, the disruption of which underlies behavioural hyperactivity after lesions of Hip or LS^15^. Our findings further show that the Hip–LS pathway mediates the stabilizing effect of hippocampal theta oscillations on locomotion. Theta-frequency stimulation of the Hip via LS-induced disinhibition activates dopaminergic neurons in the ventral tegmental area and this pathway supports reinstatement of reward memories by environmental context^16^. Here we show that theta-rhythmic activation of LS projections to the LH decreases running speed. The LH is crucial for control of locomotion^5^,^17^,^18^,^60^ and arousal^61^. The joint regulation of locomotion and cortical state has been also shown for mesencephalic locomotor region^62^. The present study shows that movement-dependent bottom-up modulation from subcortical regions to the Hip is complemented by the top-down feedback, signalled by the Hip to locomotor circuits. Our findings further suggest that hippocampal theta rhythmic signalling is read out in parallel by cortical and subcortical regions, rapidly regulating exploratory activity according to representations of the environment.

## Methods

### Experimental subjects

*PV-Cre* knock-in mice (The Jackson Laboratory, Bar Harbor, Maine, USA)^63^, *Vgat-ires-Cre* (The Jackson Laboratory)^64^ knock-in mice and *C57BL/6* male mice, 10–25 weeks old, were used. Mice were housed under standard conditions in the animal facility and kept on a 12 h light/dark cycle. All procedures were performed in accordance with national and international guidelines, and were approved by the local health authority (Landesamt für Gesundheit und Soziales, Berlin).

### Virus injections

For virus injections^65^, animals were anaesthetized with isoflurane and placed in a stereotaxic head frame (David Kopf Instruments, Tujunga, CA, USA). After a midline incision, a craniotomy was made using a dental drill. A 34-gauge bevelled metal needle connected via a tube with a microsyringe pump (PHD Ultra, Harvard Apparatus, Holliston, MA, USA) was used to infuse viruses at a rate of 100 nl min^−1^. Following infusion, the needle was kept at the injection site for 10 min and was then slowly withdrawn before the incision was sutured. Optogenetic constructs from Dr Karl Deisseroth and Dr Scott Sternson, purchased from Penn Vector Core (Philadelphia, PA, USA) or UNC Gene Therapy Center Vector Core (Chapel Hill, NC, USA) were used. *PV-Cre* mice (The Jackson Laboratories) were injected in the MS (AP 0.98, L 0.0, V −5.0 and −4.5 mm) with a total of 1 μl of Cre-dependent ChR2 (*AAV2/1.CAGGS.flex.ChR2.tdTomato.WPRE.SV40*, Penn Vector Core). For experiments using halorhodopsin (eNpHR3.0), *PV-Cre* mice were additionally injected bilaterally in the Hip (AP −1.7, L ±1.05, V −2.05 and −1.4 mm; AP −1.7, L ±1.7, V −2.05 and −1.55 mm; AP −2.3, L ±1.5, V −2.2 and −1.3 mm; AP −2.3, L ±2.2, V −1.65 and −2.45 mm) with a total of 2.4 μl of *AAV2/1.CamKIIa.eNpHR3.0-EYFP.WPRE.hGH* (Penn Vector Core). For experiments using DREADDs^66^, *PV-Cre* mice were injected in the MS with Cre-dependent ChR2 as described above and in the Hip with a total of 2 μl of *AAV8.CaMKIIa.hM4D*(Gi).mCherry (construct from Dr Bryan Roth, UNC Gene Therapy Center Vector Core). For manipulations of the LS–LH pathway, *Vgat-Cre* mice were injected bilaterally in the LS (right: AP 0.74, L 0.38, V: 3.3 mm; left: AP 0.62, L 0.33, V 3.45 mm) with a total of 1.2 μl of *AAV2/5.Ef1a.DIO.ChETA(E123T/H13R).EYFP.WPRE.hGH* (Penn Vector Core).

### Stereotaxic implantations

Optic fibre implants were fabricated from 100 μm diameter fibre (0.22 NA, Thorlabs, Newton, NJ, USA) and zirconia ferrules (Precision Fiber Products, Milpitas, CA, USA). For optogenetic entrainment of hippocampal theta oscillations, mice were unilaterally (or bilaterally in contralateral entrainment experiments, 7 mice) implanted with optic fiber implants in the CA1 str. pyramidale (AP −1.94, L 1.4, V −1.4 mm, 21 mice). For optogenetic inhibition of hippocampal projections to the LS, optical fibers were bilaterally implanted on top of the LS (AP −0.1, L −0.25, V 2.25 mm and AP 0.5, L 0.3, V 2.7 mm, 8 mice). For optogenetic stimulation of LS projections to the LH, optical fibers were bilaterally implanted above the LH (AP −1.6, L 1, V 4.8 mm, 6 mice). For inhibition of hippocampal projections to the LS using DREADDs, guide cannulas (22 gauge, PlasticsOne, Roanoke, VA, USA) were implanted bilaterally (4 mice) or unilaterally (2 mice) above the LS as described above.

Arrays of single tungsten wires (40 μm, angular cut, California Fine Wire Company, Grover Beach, CA, USA), microdrives (Minidrive-8, BioSignal Group, New York, USA) loaded with 8 tetrodes (independently movable, fabricated from 12 μm tungsten wire, California Fine Wire Company), linear silicon probes (CM32, NeuroNexus Technologies, Ann Arbor, MI, USA) or octrode probes (B32 and B64, NeuroNexus Technologies) mounted on a custom-made microdrive were used. These were implanted either in the Hip (AP −1.94, L 1.4, V −1.4 mm, wire arrays, 17 mice), or above the Hip with subsequent positioning in the CA1 pyramidal cell layer (tetrodes, 1 mouse, B32 probes, 3 mice), using LFP and unitary activity as a reference. The following coordinates were used for electrode implantations in the LS: AP 0–0.5, L 0.2–0.45, V 2.3–3.4 mm (a wire array, 1 mouse; CM32, 2 mice; B32 and B64 probes, 2 mice), complemented by a wire array implanted in the dorsal Hip (AP 2.1, L 1.6, V 1.5 mm) in 3 mice. Coordinates used for implantation of a B32 probe in the LH were: AP −1.58, L 1, V 5 mm (1 mouse). Reference and ground electrodes were miniature stainless-steel screws in the skull above the cerebellum. The implants were secured on the skull with dental acrylic.

### Data acquisition

Electrodes were connected to operational amplifiers (HS-8, Neuralynx, Bozeman, Montana USA or Noted B.T., Pecs, Hungary) to eliminate cable movement artifacts. Electrophysiological signals were differentially amplified, band-pass filtered (1 Hz–10 kHz, Digital Lynx, Neuralynx) and acquired continuously at 32 kHz. A light-emitting diode was attached to the headset to track the animal's position (at 25 Hz) during exploration. Timestamps of laser pulses were recorded together with electrophysiological signals.

### Opto- and chemogenetic stimulation

For experiments using optogenetic stimulation, a 3 m long fiberoptic patch cord with protective tubing (Thorlabs) was connected to a chronically implanted optical fiber with a zirconia sleeve (Precision Fiber Products), which allowed the mice to freely explore an enclosure (a rectangular box, 48 × 30 cm) during optogenetic stimulation. The patch cord was connected to a 473-nm diode-pumped solid-state laser (R471005FX, Laserglow Technologies, Toronto, ON, Canada) with an FC/PC adapter. The laser output was controlled using a stimulus generator and MC_Stimulus software (Multichannel Systems, Reutlingen, Germany). Optogenetic stimulation of MS–Hip projections consisted of 30 ms blue (473 nm) light pulses with light power output of 5–10 mW from the tip of the patch cord measured with a power meter (PM100D, Thorlabs). Subjects were randomly assigned to the experimental conditions. Optogenetic stimulation started at least 2 min after a baseline exploratory epoch and was preceded by ∼20 s of running or immobility. For the characterization of optogenetic theta entrainment, blue light was delivered at 7 or 10 Hz for 2 min or at 2, 4, 6, 8, 10 and 12 Hz for 45 s or at 2, 9 and 20 Hz for 1 min each. To study the effect of non-rhythmical stimulation, 1-s pulses of blue light were delivered every 3.7 s for a total of 2 min. In experiments that combined optogenetic theta entrainment and bilateral optogenetic inhibition, LS optic fibre implants were connected via patch cords to a 593-nm diode-pumped solid-state laser (R591005FX, Laserglow Technologies) using a multimode fibre optic coupler (FCMM50-50A-FC, Thorlabs). Continuous yellow (593 nm) light, ∼20 mW from the tip of each patch cord, was delivered to the LS, whereas blue light was delivered at 7 or 9 Hz to the Hip. During control light stimulation, optic patch cords were connected to dummy ferrules, attached to the headset and light of the same wavelength, frequency and power as during opsin-activating stimulation was delivered. In an additional set of controls for optogenetic inhibition experiments, light was delivered to the LS of mice expressing fluorophore (mCherry) but not eNpHR3.0.

For chemogenetic inhibition of the Hip–LS pathway, CNO (Sigma-Aldrich, St Louis, MO, USA) (100 μM, 0.3 μl) or vehicle was infused via an injector (28 gauge), which protruded 1 mm from the tip of the guide cannula using a microsyringe pump (PHD Ultra, Harvard Apparatus) at a rate of 100 nl min^−1^, while the mouse was resting in the home cage. The injector was left in place for additional 2 min and was then slowly withdrawn.

Bilateral optogenetic stimulation of GABAergic LS axon terminals in LH with 473 nm blue light consisted of 30-ms pulses at 9 Hz with light power output of 10–25 mW from the tips of the optic fibres as mice freely explored an enclosure as described above.

### Histology

After completion of the experiments, mice were deeply anaesthetized and electrolytic lesions at selected recording sites were performed. Subsequently, the animals were perfused intracardially with 4% paraformaldehyde solution and decapitated. Brains were fixed overnight in 4% paraformaldehyde, equilibrated in 1% PBS for an additional night and finally cut in 40-μm slices using an oscillating tissue slicer (EMS 4500, Electron Microscopy Science, Hatfield, PA, USA). Brain slices were mounted (Flouromount Aqueous Mounting Medium, Sigma-Aldrich). Images were taken using an Olympus BX 61 microscope (× 2/0.06 numerical aperture (NA), × 10/0.3 NA and × 20/0.5 NA, dry) or using a Leica DM 2500 microscope (× 20/0.7 NA, oil-immersion objective; Fig. 1a(1), Fig. 4e (lower right corner) and Fig.4h (lower right corner)).

### Signal processing and data analysis

Electrophysiological signals and position tracking data were processed using Neurophysiological Data Manager (NDManager^67^, http://neurosuite.sourceforge.net/). LFP was obtained by low-pass filtering and down-sampling of the wide-band signal to 1,250 Hz. In each recording, a channel with the maximal amplitude of theta oscillations was selected. Further data processing was performed by custom-written MATLAB (Mathworks, Natick, MA, USA) algorithms^19^,^29^. Data analysis was performed using automatic selection of data from the database. Sample size was estimated using http://biostat.mc.vanderbilt.edu/wiki/Main/PowerSampleSize software. Laser pulse timestamps and borders of stimulation epochs were detected. Power spectral density (PSD) was computed for each 10 s LFP epoch using the multitaper method (the time-halfbandwidth product 3, window size 8,192). The fidelity of optogenetic theta entrainment was quantified as the ratio of cumulative PSD around the optogenetic stimulation frequency (±0.5 Hz) to the cumulative PSD in the 5–12 Hz band. Recording epochs with the dominant PSD peak ≤5 Hz were excluded from analysis.

Phase-amplitude coupling of theta and gamma oscillations was assessed for epochs of theta oscillations with the theta/delta power ratio of at least 6 (ref. 29). Theta phase was obtained by the Hilbert transformation of 5–10 Hz filtered signal. Gamma oscillation peaks were detected in the 35–85 Hz band-pass filtered signal and their amplitudes and theta phases were computed. The modulation coefficient^19^ was computed for each theta/delta ratio-standardized theta amplitude bin.

Action potentials were detected in a band-pass filtered signal (0.8–5 kHz). Events with a magnitude exceeding 3 s.d. above mean signal magnitude were detected, spike waveforms were extracted and represented by the first three principle components. Spike sorting was performed automatically followed by manual clusters adjustment^68^. Putative pyramidal cells were identified based on their auto-correlograms and firing rate (<3.5 Hz). Single pyramidal cells with a clear refractory period (≤2 ms) as well as single and multiunit neurons recorded in LS and LH (mean isolation distance 61±4 and 54±4, respectively) were used in further analysis.

Theta oscillation phase was extracted by linear interpolation between wave peaks and troughs^29^. Phase histograms of individual spike trains and of laser pulse trains were computed and normalized, first, by the deviation (if any) of the underlying phase distribution from uniformity in respective phase bins^69^ and, second, by the total number of events. Circular uniformity (Rayleigh test), mean phase and the resultant vector length were estimated for each histogram. Histograms of preferred discharge phases were computed by grouping mean phases of individual cells. Individual unit discharge probability histograms were convolved with the Gaussian kernel^20^ of size 2 s.d.

For correlation of firing probability changes across theta cycles with differences of theta cycle amplitudes, each spike was assigned with the index of the concurrent theta cycle, the respective theta cycle amplitude and the phase bin (±30° from theta peak or trough), where the spike was emitted. Spikes emitted outside these phase bins and neurons, which fired less than ten spikes per phase bin during a recording session, were excluded from analysis. Pair-wise difference of spike count (S) within bins and differences of theta cycles amplitude (A), defined as:


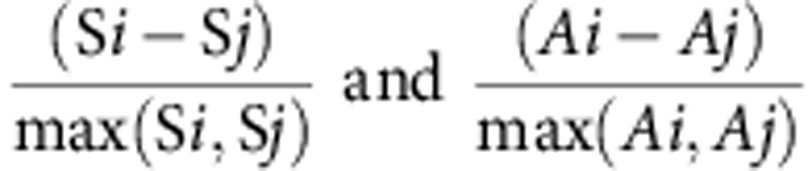


respectively, where *i* and *j* are indexes of theta cycles, were computed for all combinations of theta cycles in each recording. Linear regression was used to measure relation between difference of spike count and difference of theta cycles amplitude for each neuron. Slopes and intercepts were estimated. Neurons with slopes significantly different from zero were used in subsequent analysis. Average differences of spike count were computed for each bin of theta amplitude differences and their absolute values were averaged across neurons.

Firing maps of pyramidal cells were computed as previously described^29^. The number of spikes in a given spatial pixel (2 × 2 cm) was divided by the time spent in this pixel. Periods of immobility were excluded from the analysis. Coherence, a measure of the local smoothness of the firing profile, was computed as the Fisher *z*-transform of the Pearson's correlation between the rate in a given pixel and the average rate in its eight first-order neighbours. Sparsity, a measure of firing field compactness was computed as in ref. 70.

Instantaneous running speed was computed from mouse positions and low-pass filtered to eliminate speed swings due to an animal's head movement^10^. Average running speed, CV of the running speed, optogenetic entrainment fidelity, duration of continuous running, fraction of time running, length of the path, average amplitude of theta peaks and the CV of the theta amplitude were computed for running (>2 cm s^−1^) within each 10-s epoch. Optogenetic stimulation epochs with the entrainment fidelity below 0.3 (<20% of recordings) were excluded from analysis, owing to insufficient optogenetic control of theta rhythm.

The statistical significance of comparisons was determined by repeated-measures analysis of variance, by F-test for comparisons of model fits or by the *χ*^2^-test for comparisons of proportions. Circular statistics were performed using Rayleigh and Watson–Williams tests. *P*-values <0.05 were considered to indicate significance.

## Additional information

**How to cite this article:** Bender, F. *et al*. Theta oscillations regulate the speed of locomotion via a hippocampus to lateral septum pathway. *Nat. Commun.* 6:8521 doi: 10.1038/ncomms9521 (2015).

## Supplementary Material

Supplementary Information Supplementary Figures 1-6, Supplementary Table 1 and Supplementary Methods
