# Supplementary material for: Theta oscillations regulate the speed of locomotion via a hippocampus to lateral septum pathway
Source: Nat Commun. 2015 Oct 12;6:8521. doi: 10.1038/ncomms9521 (PMC4633825; doi:10.1038/ncomms9521)
Supplement: Supplementary Information — Supplementary Figures 1-6, Supplementary Table 1 and Supplementary Methods [file ncomms9521-s1.pdf]

Supplementary Figure 1

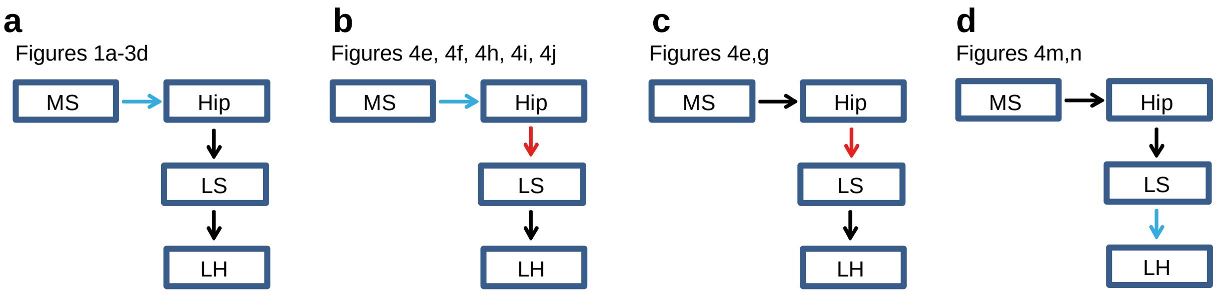

Supplementary Figure 1. Experimental design.

(**a**) In experiments shown in Fig. 1a-3d, an excitatory opsin, channelrhodopsin2 (ChR2) was expressed in GABAergic (PV-positive) cells of the medial septum (MS), while an optic fiber was implanted above the CA1 hippocampal area (Hip). Blue light, which activates ChR2, was delivered via the optic fiber to stimulate MS-Hip projections. (**b**) In experiments shown in Fig. 4e, 4f, 4h, 4i and 4j, in addition to the expression of ChR2 in the MS as described in the previous scheme, inhibitory DREADDs (Fig. 4f) or an inhibitory opsin (halorhodopsin, eNpHR3.0) (Fig. 4h-j) was expressed in Hip. When DREADDs were used (Fig. 4f), CNO, the DREADDs-activating compound, or vehicle, was injected in lateral septum (LS) to inhibit Hip-LS projections. In addition, an optic fiber was implanted above hippocampus and blue light was delivered to stimulate MS-Hip projections, i.e. to entrain theta as in experiments shown on Fig. 1a-3d. For experiments where halorhodopsin was expressed in hippocampus (Fig. 4h-j), yellow light, which activated halorhodopsin, was delivered via the optic fibers implanted bilaterally above LS to inhibit Hip-LS projections. For the additional illustration of this preparation see also Supplementary Fig. 6i. (**c**) In experiments shown on Fig. 4g, theta oscillations were not optogenetically entrained, i.e. blue light was not switched on. Inhibitory DREADDs were expressed in Hip, while CNO, or vehicle, was injected in LS to inhibit Hip-LS projections. (**d**) In experiments shown in Fig. 4m and 4n, an excitatory opsin, ChETA was expressed in the GABAergic cells of the LS while an optic fiber was implanted above lateral hypothalamus (LH). Delivery of blue light to LH activated ChETA to stimulate GABAergic LS-LH projections.

Supplementary Figure 2

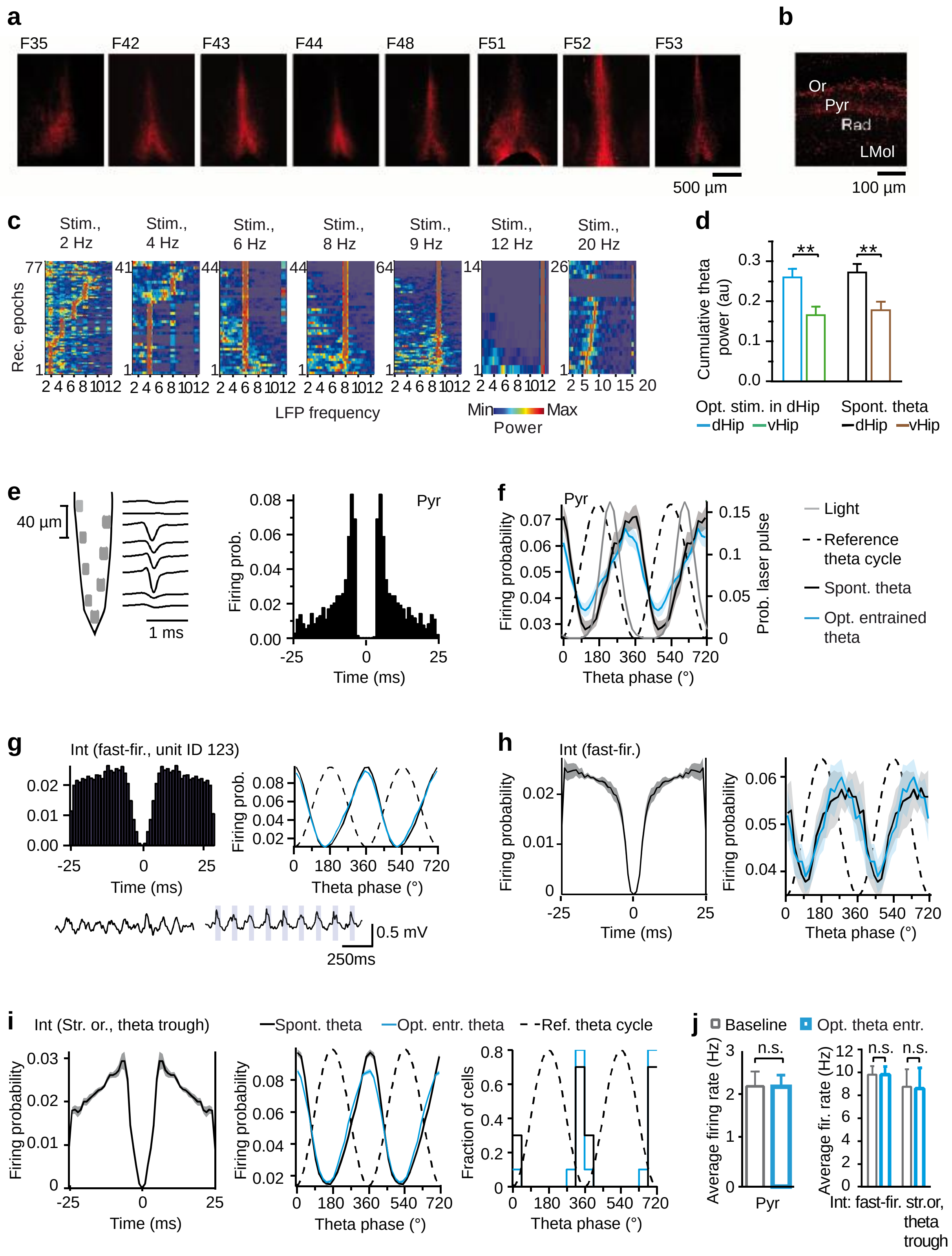

Supplementary Figure 2. Optogenetic control of hippocampal theta oscillations.

(**a**) Channelrhodopsin2 expression in separate *PV*-Cre mice with *AAV2/1.CAGGS.flex.ChR2.tdTomato.WPRESV40* injections in the medial septum.
(**b**) Expression of ChR2 in hippocampal axons of MS neurons. MS-Hip projections terminate preferentially in str. oriens, followed by str. radiatum and str. lacunosum-moleculare. Or - str.oriens, Pyr - str.pyramidale, Rad - str. radiatum, LMol - str. lacunosum-moleculare.
(**c**) Distribution of LFP power (color axis) in various recording epochs during optostimulation at different frequencies. Note that optogenetic stimulation is most efficient within the theta frequency band (6-12 Hz), individual power spectra (rows) in these plots were ordered according to entrainment fidelity. During stimulation at 4 Hz hippocampal oscillations were entrained at the stimulation frequency only in ~63% of recording epochs, during stimulation at 2 Hz - only in ~29% (*N*=9 mice). For stimulation frequencies outside the theta band (2, 4, 20 Hz), rows were ordered according to frequency of the dominant power spectral peak.
(**d**) Cumulative theta power in dorsal (dHip) and intermediate-ventral (vHip) hippocampus during spontaneous theta (black and brown bars) and optogenetic entrainment of dHip theta (blue and green bars). Theta power was higher in dHip than in vHip both during optogenetic dHip entrainment (p=0.0063) and during spontaneous theta oscillations (p=0.0063). Optogenetic entrainment affected neither this power relation ($F_{1,15}$=0.25, p=0.622) nor coherence between dHip and vHip (p=0.31, signed rank test). See also Supplementary Fig. 5.
(**e**) An example of an isolated single CA1 pyramidal cell. Average spike waveforms (middle) recorded using a silicon probe, which image is shown to the left, and the corresponding auto-correlogram (right). Bin width, 1 ms. (**f**) Rhythmic changes of pyramidal cells' firing probability during spontaneous (Pyr, black, *n*=29 neurons) and optogenetically entrained (blue, n=30 neurons) theta oscillations. Black dotted line - reference oscillation cycle. Preferred discharge phases were not significantly different (p=0.79). (**g**) Auto-correlogram of a fast-firing interneuron (Int. (fast-fir.), firing rate 17 Hz) (left) and rhythmic changes of its firing probability (right) during spontaneous (black line) and optogenetically entrained (blue line) theta oscillations. Below: LFP traces showing spontaneous (left) and optogenetically entrained (right) theta oscillations from this experiment. (**h**) Average auto-correlogram (left) and changes of firing probability of fast-firing interneurons during spontaneous (black) and optogenetically entrained (blue) theta oscillations (right, *n*=28 neurons). In both conditions fast-firing interneurons discharged preferentially during the descending part of the theta cycle (preferred phases, p=0.97). (**i**) Average auto-correlogram of neurons recorded from str. oriens, which preferentially fired close to the theta trough during baseline recordings (Int (Str. or., theta trough), left). Changes of firing probability (middle) and histograms of preferred discharge phases (right) during spontaneous (black) and optogenetically entrained (blue) theta oscillations (preferred phases: p=0.56, *n*=10 neurons). Criteria for assignment to this group were derived from recordings of identified oriens-lacunosum moleculare (O-LM) cells[21,22]: these units were recorded in CA1 str. oriens (based on the electrodes' positions, oscillatory LFP landmarks and absence of concurrent pyramidal cell discharge[20]), had their preferred firing phase within ±30 degrees from the theta trough during baseline recordings, typical for interneurons distributions of interspike intervals (assessed by auto-correlations, left plot) and average firing rates above 3 Hz. (**j**) Average firing rates of hippocampal pyramidal cells (p=0.98, *n*=11), fast-firing interneurons (p=0.96, fast-fir., *n*=28) and str. oriens, theta trough interneurons (p=0.85, str. or., theta trough, *n*=10), before and during optogenetic entrainment at 7 Hz. Data are presented as mean±s.e.m.

Supplementary Figure 3

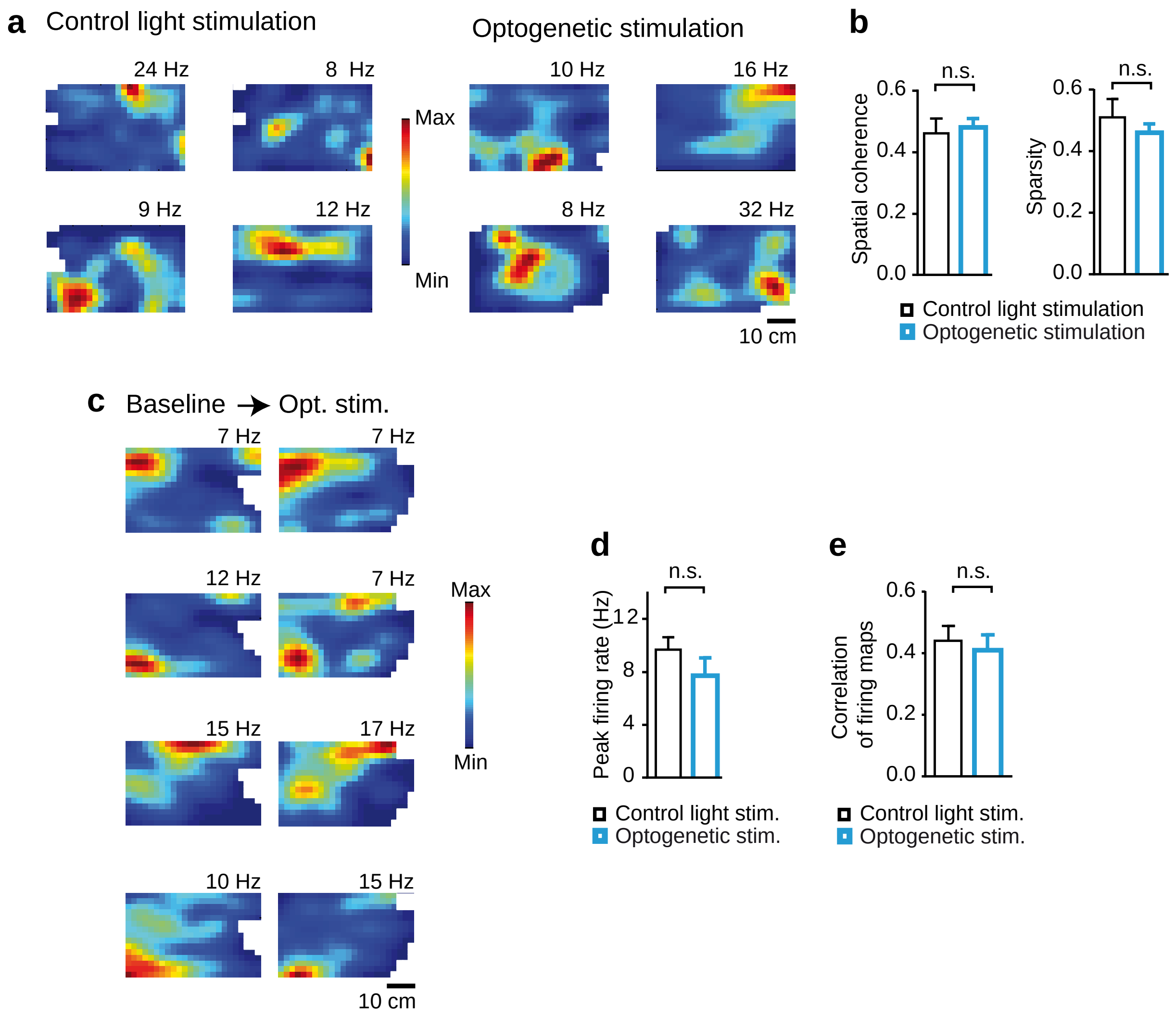

Supplementary Figure 3. Spatial firing properties of CA1 pyramidal cells during spontaneous and optogenetically entrained theta oscillations.

(**a**) Representative firing maps of pyramidal cells recorded in the CA1 area during exploration of a rectangular enclosure, accompanied by spontaneous theta (during control light stimulation, left) or optogenetically entrained at 7 Hz theta oscillations (right). The firing rate of individual cells at each particular location is colour-coded (linear scale from zero to max shown in center). The peak firing rate (maximum of each firing map) is shown in the right upper corner of each map. (**b**) Spatial coherence and sparsity (left, coherence, *p*=0.69; right, sparsity, *p*=0.41; *n*=22 cells, spontaneous theta, *n*=51 cells, optogenetic stimulation, *t*-test, *N*=4 mice) did not differ between control light and optogenetic stimulation. (**c**) Firing maps computed for the same CA1 pyramidal cells during baseline, followed by optogenetic stimulation. (**d**) Peak firing rate (*p*=0.10, *n*=18 cells, *N*=4 mice) did not change during optogenetic stimulation, in comparison to control light stimulation in the same pyramidal cells. (**e**) Correlations of firing rate in the same spatial locations did not differ across two control recording sessions compared to control vs. optogenetic stimulation sessions (*p*=0.51, *n*=14 cells, control; *n*=18 cells, optogenetic stimulation). Data are presented as mean±s.e.m.

Supplementary Figure 4

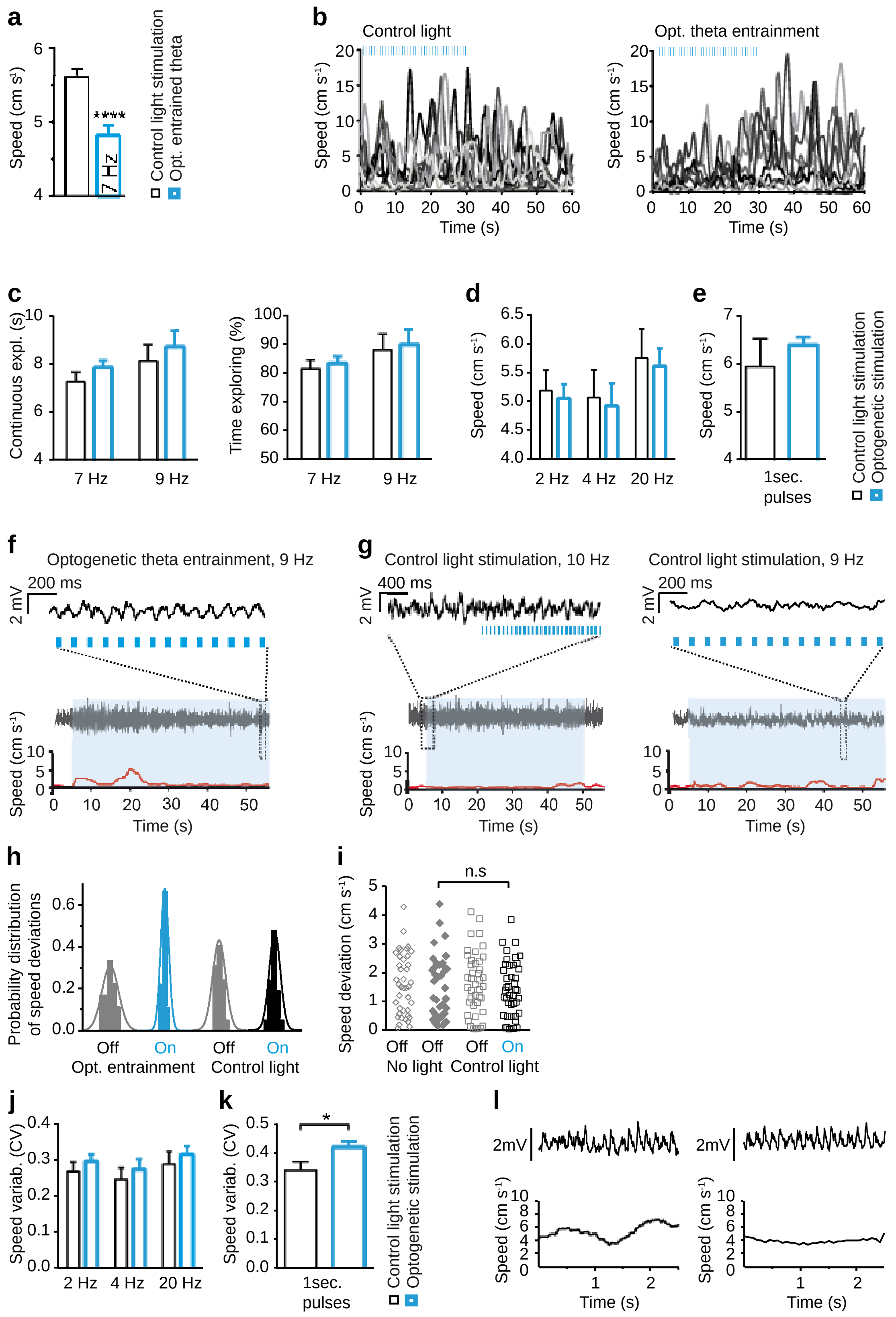

Supplementary Figure 4. Hippocampal theta oscillations regulate running speed and its variability.

(**a**) Running speed during optogenetic theta entrainment (at 7 Hz, blue) and epochs of 5-10 Hz theta oscillations during control light stimulation (black) (p=0.000019, N=5 mice).
(**b**) Representative speed traces during 30 seconds of theta entrainment (fidelity>0.3, n=10 traces, N=5 mice) or control light (n=10 traces, N=8 mice) at 10 Hz and additional 30 seconds after the light was switched off. (**c**) Time of continuous exploration (left, p=0.97) and total time exploring (right, p=0.54) during optogenetic theta entrainment and epochs of 5-10 Hz theta oscillations during control light stimulation. (**d**) Running speed during 2 Hz (N=7 mice, n=47 recording epochs), 4 Hz (N=5 mice, n=19) or 20 Hz (N=5 mice, n=29) optogenetic stimulation compared to control light stimulation ($F_{2,84}$=0.07, p=0.8). Experiments with average baseline running speed >5 cm s^{-1} are shown to facilitate comparison with Fig. 2c. Speed values also did not differ between optogenetic and control light stimulation for the overall running speed, i.e. >2 cm s^{-1} ($F_{1,131}$=0.31, p=0.58): 2 Hz (opt. entr.: 4.57±0.26 cm s^{-1} vs. baseline: 4.37±0.34 cm s^{-1}; N=8 mice, n=73), 4 Hz (opt. entr.: 4.43±0.38 cm s^{-1} vs. baseline: 4.23±0.42 cm $s^{-1;}$ N=6 mice, n=28) or 20 Hz (opt. entr.: 4.86±0.32 cm s^{-1} vs. baseline: 4.67±0.46 cm s^{-1}; N=6 mice, n=43). (**e**) Running speed during optogenetic stimulation with 1 second continuous light pulses (N=10 mice) compared to control light stimulation (N=9 mice, p=0.29). (**f**) Examples of recording sessions where optogenetic stimulation at 9 Hz elicited theta but not movement in a resting mouse. Theta oscillations were observed during the whole stimulation trial. Hippocampal LFP signal traces (2-250 Hz band-pass filtered, middle), recorded simultaneously with running speed measurement (red trace, bottom). Blue shade marks time of optostimulation. Excerpt, top: LFP signal trace shown at a higher time resolution. (**g**) Running speed before and after onset of control light stimulation, which did not evoke hippocampal theta oscillations. Black, dashed rectangles show magnification of LFP signal traces. (**h**) Histogram showing the distributions of data presented on main Figure 2g. (**i**) Speed (deviation from group mean) averaged for 20 sec during spontaneous locomotion in the darkness, i.e. without light delivery during the first (open grey diamonds) or the second (filled grey diamonds) 20 sec epochs, or before (grey squares) and during (black squares) control light stimulation. Speed deviation did not differ between control light onset and spontaneous locomotion in darkness (p=0.75, N=13 mice).
(**j**) Speed variability (CV) during optogenetic stimulation at 2 Hz (N=8 mice), 4 Hz (N=6 mice) or 20 Hz (N=6 mice) compared to control light stimulation at respective frequencies (p=0.51).
(**k**) Speed variability (CV) during optogenetic stimulation with 1 second continuous light pulses (N=10 mice) compared to control light stimulation (p=0.047, N=9 mice). (l) Example speed traces recorded during spontaneous locomotion (without optogenetic stimulation) illustrate the association of high theta amplitude variability with high speed variability (left) as well as low theta amplitude variability with low speed variability (right). *p<0.05, ****p<0.0001. Data are presented as mean±s.e.m. See SI for details of statistical analysis.

Supplementary Figure 5

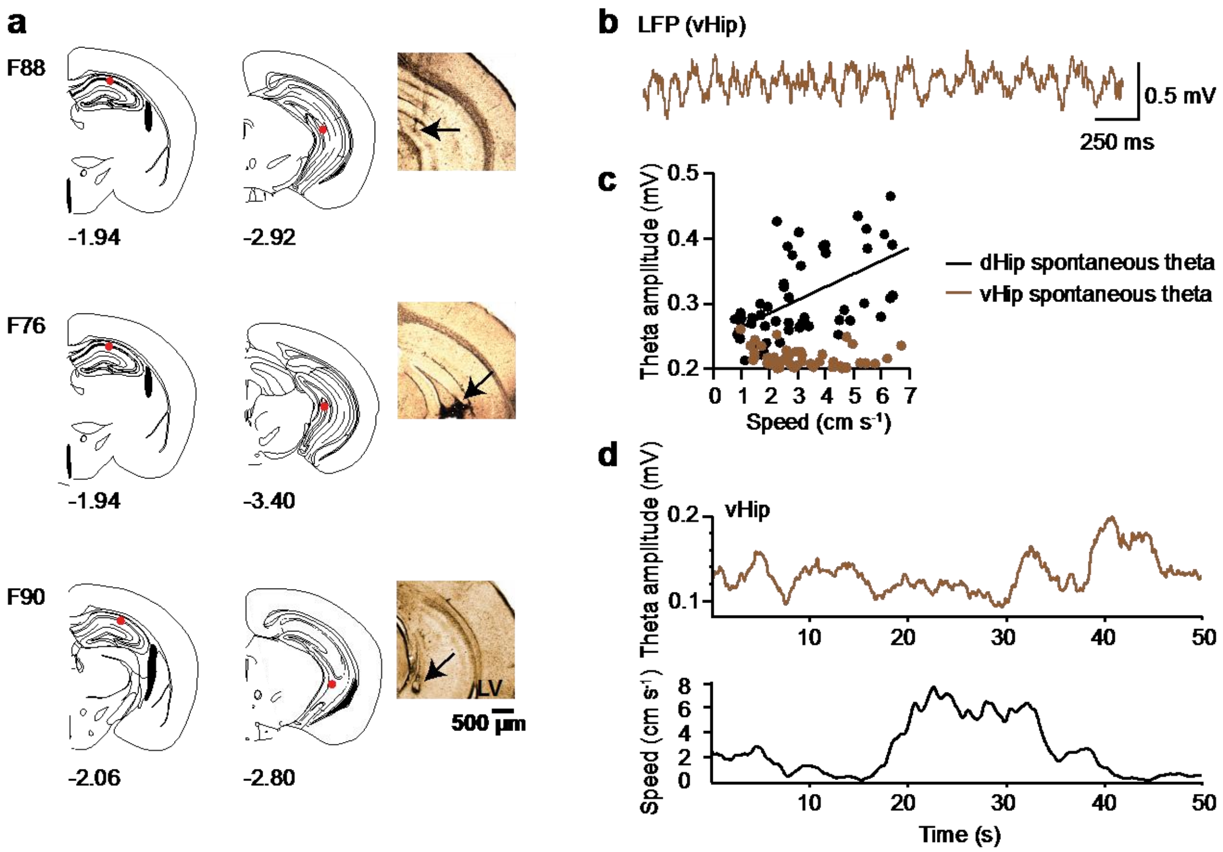

Supplementary Figure 5. LFP recordings from dorsal hippocampus (dHip) and intermediate-ventral hippocampus (vHip) during spontaneous and optogenetically entrained theta.

(**a**) Histologically verified recording sites in dHip and vHip for each mouse are indicated by red dots. Tracks of tungsten wires in the vHip are shown on the right. (**b**) LFP signal (1-200 Hz band-pass filtered) showing theta oscillations in vHip. (**c**) Theta oscillations amplitude in the dHip (5-10 Hz bandpass filtered, amplitude derived from Hilbert transform, black, r=0.59, p<0.0001), but not in a more ventral part of hippocampus (vHip, brown, r=-0.12, p=0.45) correlated with speed of locomotion. Pearson's correlation. (**d**) Representative examples of theta amplitude (upper trace, processed as in (**c**)) and speed (lower trace) showing dissociation of locomotion and theta oscillations in vHip.

Supplementary Figure 6

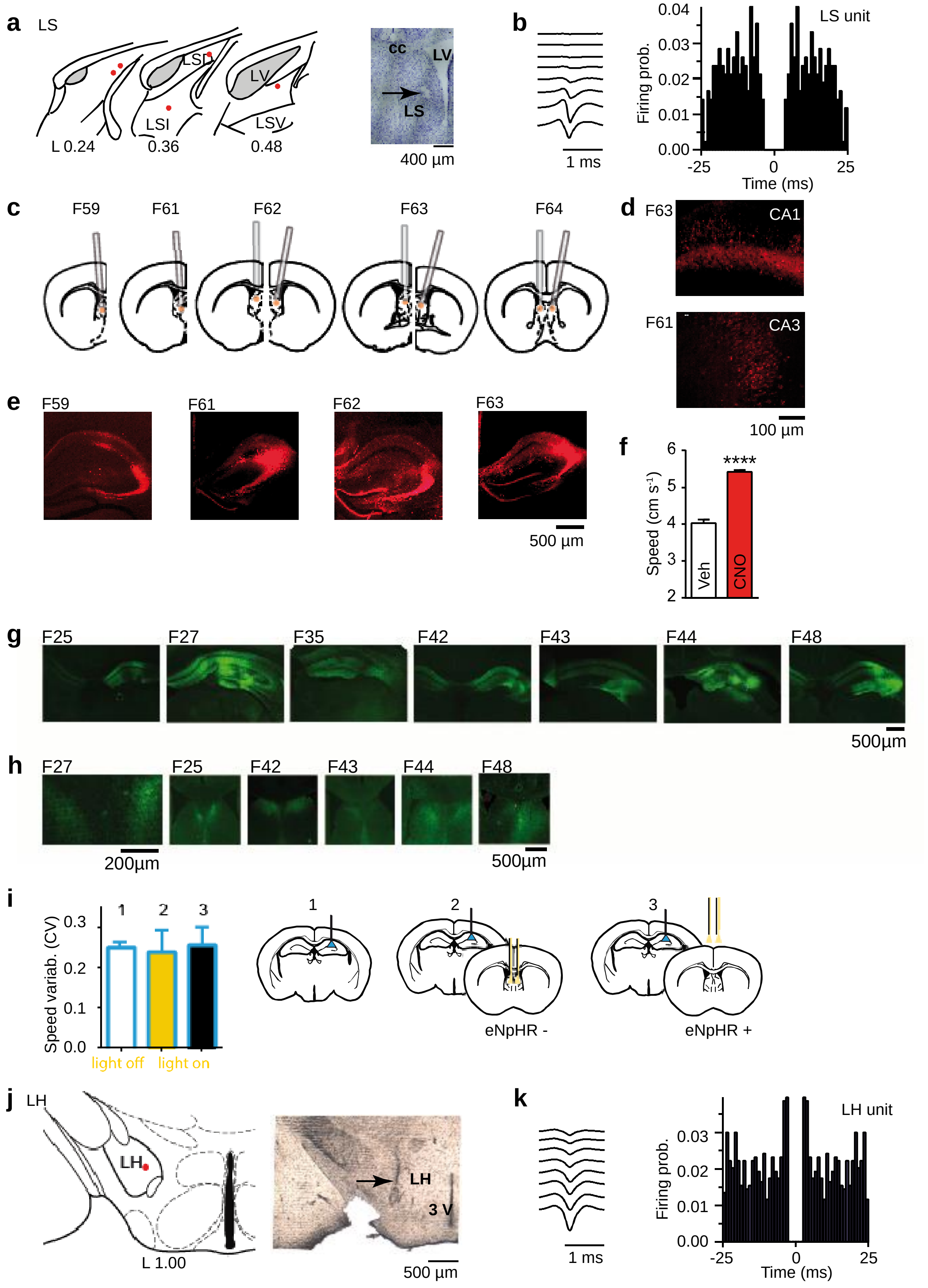

Supplementary Figure 6. Hippocampus - LS - LH pathway mediates theta-rhythmic control of locomotion.

(**a**) Positions of recording electrodes in the LS, red dots show histologically verified electrode localizations in 5 mice. Right: an example of a Nissl-stained slice with the electrolytic lesion (arrow); L 0.24 - 0.48 - distance to midline, mm, cc - corpus callosum, LSD - dorsal LS, LSI - intermediate LS, LSV- ventral LS, LV - lateral ventricle (brain images adapted from the mouse brain atlas by Paxinos and Franklin, 2013). (**b**) An example of an isolated single LS unit. Average spike waveforms recorded using a silicon probe and the corresponding auto-correlogram. Bin width, 1 ms. (**c**) Positions of microinjectors for intracranial microinjection of CNO or vehicle (separate mice). (**d,e**) hM4D(Gi) expression in animals with *AAV-CaMKIIa-hM4D(Gi)-mCherry* injection in the hippocampus. (**f**) Running speed after intra-LS CNO during epochs of spontaneous theta oscillations for overall running speed data (>2 cm s^{-1}, p<0.00001, N=6 mice). (**g,h**) Expression of eNpHR3.0 in mice with *AAV2/1.CamKIIa.eNpHR3.0-EYFP.WPRE.hGH* injections in the hippocampus.
(**g**) Expression pattern in the hippocampus. (**h**) Immunofluorescence in LS showing eNpHR3.0 targeting to axons of hippocampal pyramidal cells. (**i**) Speed variability during ChR2-mediated MS-Hip optical theta pacing did not differ between groups of (1) mice not injected with eNpHR3.0 virus in hippocampus (eNpHR3.0-), LS yellow (593 nm) light off, (2) mice expressing mCherry in hippocampus-LS (eNpHR3.0-), LS yellow light on, (3) mice expressing eNpHR3.0 (eNpHR3.0+), during optogenetically entrained theta, yellow light was delivered outside the brain (control light stimulation) (p=0.96, N=13 mice). (**j**) Position of the silicon probe in the LH and the silicon probe track (right). (**k**) An example of an isolated LH unit. Average spike waveforms recorded using a silicon probe and the corresponding auto-correlogram. Bin width, 1 ms. See Supplementary Information for details of statistical analysis. Data are presented as mean±s.e.m.

Supplementary Table 1

**Speed**	Confound		
Predictor	θ Freq. variability	θ Amplitude	θ Ampl. var. (CV)
Entrainment fidelity (estimate of θ Freq. regularity)		r = - 0.129<br>p = 0.008	r = - 0.027<br>p = 0.584
θ Amplitude	r = 0.092<br>p = 0.059		r = 0.061<br>p = 0.208
θ Ampl. variability (CV)	r = 0.213<br>p = 0.0001	r = 0.237<br>p < 0.00001	
**Speed variability**	Confound		
Predictor	θ Freq. variability	θ Amplitude	θ Ampl. var. (CV)
Entrainment fidelity (estimate of θ Freq. regularity)		r = - 0.146<br>p = 0.003	r = - 0.101<br>p = 0.0392
θ Amplitude	r = 0.040<br>p = 0.405		r = - 0.009<br>p = 0.847
θ Ampl. variability (CV)	r = 0.105<br>p = 0.031	r = 0.144<br>p = 0.003	

Supplementary Table 1. Direct impact of theta oscillations on locomotion.

Partial Pearson correlations (and respective p-values) of optogenetically entrained theta oscillations‘ parameters with running speed and its variability, adjusted for the influence of other confounding theta oscillation parameters (*N*=9 mice, *n*=422 recording epochs).

Supplementary Methods

Statistical analysis

Fig. 1d: Theta amplitude-adjusted average coefficients of gamma amplitude modulation were not different between spontaneous (*n*=8) and optogenetically entrained (*n*=19) hippocampal theta LFP recordings (*F*-test, $F_{4,8}$=1.04, *p*=0.42, *N*=3 mice). Polynomial model fits:

$y = -0.0005x^3 + 0.02x^2 - 0.16x + 0.79$, R^2=0.94 (spontaneous theta),

$y = -0.0002x^3 + 0.008x^2 - 0.06x + 0.52$, R^2=0.93, (optogenetically entrained theta), where *x* is theta amplitude and *y* is the coefficient of gamma amplitude modulation.

Fig. 1e: During optogenetic theta frequency stimulation, stimulation frequency (6, 8 and 10 Hz) was correlated in 5 out of 6 mice with the dominant frequency of theta oscillations recorded in the contralateral CA1 area (Pearson's correlation, *r*=0.99±0.0004, *p*<0.05 for each of the 5 mice).

Fig. 1f: Preferred theta phases of single pyramidal cells recorded during baseline spontaneous theta epochs and subsequent optogenetically entrained theta epochs did not significantly differ (Watson-Williams test, $F_{1,57}$=0.08, *p*=0.79, *n*=30 neurons, 29 of them recorded during spontaneous and 30 during optogenetically entrained theta): the preferred phase preceded the theta trough by ~40 degrees. Preferred theta phases of single- and multi-unit putative fast spiking interneurons, recorded during baseline spontaneous theta epochs and subsequent optogenetically entrained theta epochs, did not differ (301±39 and 324±37 degrees, Watson U2 permutation test, *p*=0.97, *n*=28 neurons recorded during both types of epochs).

Fig. 2a: Spontaneous hippocampal theta was correlated with running speed during spontaneous theta epochs which occurred during control 473 nm light delivery (Pearson's correlation, *r*=0.82, *p*=0.013, *n*=42 recording sessions, *N*=6 mice), but was not correlated with running speed during optogenetically entrained hippocampal theta (Pearson's correlation, *r*=0.04, *p*=0.7, *n*=72 recording sessions, *N*=8 mice).

Fig. 2c: Running speed during epochs of optogenetically entrained hippocampal theta and frequency-matched epochs of spontaneous hippocampal theta during control light stimulation. Repeated measures ANOVA, with factors "experimental subject", "type of optical stimulation (intra-Hip theta-entraining stimulation vs. control blue light stimulation)" and "stimulation frequency", revealed a significant decrease in running speed during hippocampal theta entrainment ($F_{1,248}$=17.85, *p*=0.00003;

optogenetic stimulation at 7 Hz: p=0.0002, N=5 mice; at 9 Hz: p=0.0002, N=3 mice, Bonferroni tests), no effect of stimulation frequency ($F_{1,248}$=0.82, p=0.37) and no interaction between type of blue light stimulation and stimulation frequency ($F_{1,248}$=0.31, p=0.58). See also Supplementary Fig. 4a for the analysis of speed during optogenetically entrained and spontaneous, not stimulation-frequency-matched, 5-10 Hz theta episodes.

Fig. 2f: The likelihood of movement initiation was comparable after onset of control light (n=8 out of 14 recording sessions, N=5 mice) or optogenetic hippocampal theta entrainment (n=4 out of 14 recording sessions, N=8 mice); the difference was not significant (χ^2test, $\chi^2(1)$=2.33, p=0.13).

Fig. 2g: Running speed variability (squared deviations of speed in individual experiments from a group mean) upon the onset of optogenetic theta entrainment. Repeated measures ANOVA, with factors “experimental subject”, “type of optical stimulation (intra-Hip vs. control blue light stimulation)” and “light (On vs Off)” revealed an interaction between type of optical stimulation and light ($F_{1,102}$=6.59, p=0.027). Since distributions were estimated to have different variances (see Supplementary Fig. 4h), the p-value represents the probability of the F-statistic in a distribution generated by ANOVA repetitions with within-subject permutation between factors' two levels (5000 permutations). Speed variance was decreased during optogenetic theta entrainment in comparison to the pre-stimulation baseline (p=0.0062, N=8 mice, n=18 recording epochs, Bonferroni tests) and control blue light stimulation (p=0.0107, N=13 mice, n=42 recording epochs). Control blue light stimulation did not change speed variance (p=0.68). The speed variance in baseline recordings did not differ (p=0.16). Experiments where mice were running during light stimulation were used, irrespective of animals’ speed before the onset of light stimulation (speed range: 0.1 cm s^{-1} to 8.1 cm s^{-1}) during "Off" condition. Pre-stimulation speed did not affect running speed during light stimulation (repeated measures, $F_{58,58}$=1.12, p=0.33).

Fig. 2i: Both speed variability and hippocampal theta amplitude variability (coefficients of variation, CV) were negatively correlated with entrainment fidelity (Pearson's correlation r=-0.84, p=0.0051 and r=-0.84, p=0.0046, respectively, n=79 recording sessions, N=8 mice).

Fig. 3b: Polynomial model fits:

$y = 123.1x^3 - 60.6x^2 + 7.07x + 0.08$, R^2=0.89 (spontaneous theta), and

$y = 569.2x^3 - 119.8x^2 + 8.7x + 0.01$, R^2=0.9, (optogenetically entrained theta), where x is theta amplitude variability and y is running speed variability (coefficient of variation). The dependence of running speed variability on theta amplitude variability did not differ for spontaneous (n=384 recording epochs, N=8 mice) and optogenetically entrained (n=818 recording epochs, N=8 mice) hippocampal theta ($F_{4,10}$=0.98, p=0.46, F-test).

Fig. 3c: Polynomial model fits:

$y = 9121.3x^3 - 2159.5x^2 + 170.8x + 1$, R^2=0.96 (spontaneous theta), and

$y = 5531.6x^3 - 1418.9x^2 + 122.5x + 1.6$, R^2=0.92 (optogenetically entrained theta), where x is theta amplitude variability and y is running speed.

The dependence of running speed on hippocampal theta amplitude variability did not differ for spontaneous (n=384 recording epochs, N=8 mice) and optogenetically entrained (n=818 recording epochs, N=8 mice) hippocampal theta ($F_{4,10}$=2.14, p=0.14, F-test).

Fig. 3d: Smaller differences in cycle to cycle hippocampal theta amplitude (x) were associated with more constant theta phase-matching discharge probability of pyramidal cells (y) in a similar way for spontaneous and optogenetically entrained hippocampal theta ($F_{3,14}$=1, p=0.42, F-test): all neurons included in the analysis showed significant Pearson correlation of x and y (p<0.05 for each of 25 out of 55 (spontaneous theta) and 12 out of 22 (optogenetically entrained theta) recorded neurons).

Polynomial model fits:

$y = 0.004x^2 + 0.006x + 2.6$, R^2=0.9 (spontaneous theta), and

$y = 0.005x^2 - 0.09x + 4.2$, R^2=0.88 (optogenetically entrained theta), where x is the difference of theta cycles amplitude and y is the difference of spike count in theta phase bins.

Fig. 4c: Data from three parallel recordings from the hippocampus and the LS in three mice.

Fig. 4d: Data from 21 LS single- and multi-units (out of 73 recorded units) with significantly non-uniform theta phase distributions (Rayleigh test, p<0.05 for each unit).

Fig. 4f: Running speed during optogenetic hippocampal theta entrainment following intra-LS CNO or vehicle administration. Two-way repeated measures ANOVA, with factors “experimental subject” and “drug treatment (intra-LS CNO or vehicle)”,

revealed that during optogenetic entrainment of hippocampal theta running speed was significantly higher following CNO administration in comparison to vehicle administration ($F_{1,18211}$=514.94, p<0.00001, N=5 mice). The running speed was not different during baseline recordings ($F_{1,39739}$=0.09, p=0.77).

Fig. 4g: Running speed following intra-LS CNO or vehicle administration. Two-way repeated measures ANOVA, with factors “experimental subject” and “drug treatment (intra-LS CNO or vehicle)”, revealed that running speed was significantly increased following CNO administration in comparison to vehicle administration both in the overall running speed data (see Supplementary Figure 6f) and in the shown on the Fig. 4g subset of recording epochs, in which average running speed following vehicle administration was similar to that in Fig. 2c, Fig. 4f,n, i.e. >5 cm s^{-1}, ($F_{1,10809}$=29.23, p<0.00001, N=6 mice). The speed variability (CV) did not significantly increase following CNO application ($F_{1,13}$=2.64, p=0.15, N=6 mice).

Fig. 4j: Running speed variability (CV) during baseline and optogenetic hippocampal theta entrainment co-applied with Hip-LS (eNpHR3.0) inhibitory stimulation. Two-way repeated measures ANOVA, with factors “experimental subject” and “type of optical stimulation (intra-LS yellow light or no yellow light stimulation)”, revealed that during hippocampal theta entrainment LS yellow light delivery significantly increased running speed variability (CV) ($F_{1,36}$=8.38, p=0.0073). The variability of the running speed during respective baseline epochs was not significantly different ($F_{1,66}$=0.48, p=0.49, N=8 mice).

Fig. 4l: Normalized average firing rate of 11, out of 17 recorded, LH neurons (single and multi-units) which displayed a significant correlation of firing rate with the running speed (Pearson's correlation coefficient, p<0.05 for each unit).

Fig. 4n: Running speed during optical theta-frequency stimulation of ChETA-expressing LS-LH projections. Repeated measures ANOVA, with factors “experimental subject”, “experimental epoch (baseline vs. stimulation)” and “type of optical stimulation (intra-LH stimulation vs. control blue light stimulation)”, revealed an interaction between light condition and type of stimulation ($F_{1,32}$=4.69, p=0.04) and a significant effect of light stimulation on running speed ($F_{1,32}$=7.05, p=0.012). Subsequent Bonferroni tests indicated that optogenetic stimulation of LS-LH projections reduced running speed (LH stimulation: p=0.003, N=7 mice, n=36 recordings; control stimulation: p=0.99, N=8 mice, n=32 recordings).

Supplementary Fig. 2d: Repeated measures ANOVA, with factors “experimental subject”, “light delivery (optogenetic dHip theta entrainment vs. light off)” and "part of hippocampus (dHip vs. vHip)", revealed a significant difference in theta power between dHip and vHip ($F_{1,15}$=14.48, p=0.0017; Tukey-Kramer post-hoc test; optogenetic dHip theta entrainment: p=0.0063, no light delivery: p=0.0063). Optogenetic entrainment affected neither this power relation ($F_{1,15}$=0.25, p=0.622) nor coherence between dHip and vHip (p=0.31, signed rank test, N=3 mice, n=5 recordings).

Supplementary Fig. 2f,h: see Fig. 1f.

Supplementary Fig. 2i: Preferred theta phases of single- and multi-unit putative oriens-lacunosum moleculare (O-LM) interneurons. Preferred discharge phases did not differ between baseline spontaneous theta epochs and subsequent optogenetically entrained theta epochs (n=10 neurons, 352±3 and 347±4 degrees prior to the theta trough, $F_{1,19}$=0.35, p=0.56, Watson-Williams test).

Supplementary Fig. 2j: Firing rates of hippocampal pyramidal cells before and during optogenetic theta entrainment at 7Hz. Two-sample t-test, t_{10}=-0.017; p=0.98, n=11 neurons. The firing rate of fast-firing interneurons (t_{27}=-0.045 p=0.96, n=28 neurons), as well as of interneurons recorded from Str. oriens, that fire during the theta trough (t_9=-0.19, p=0.85, n=10 neurons), was also not different before and during optogenetic theta entrainment at 7Hz (paired sample t-test).

Supplementary Fig. 3b: Intact spatial firing properties of CA1 pyramidal cells during spontaneous and optogenetically entrained theta oscillations: spatial coherence (t_{71}=-0.40, p=0.69, t-test, single units: n=22, spontaneous theta, n=51, optogenetic stimulation, N=4 mice) and spatial sparsity (t_{71}=0.83, p=0.41; single units: n=22, spontaneous theta, n=51, optogenetic stimulation, N=4 mice).

Supplementary Fig. 3d: Individual pyramidal cells did not change their peak firing rates during control light vs. optogenetic stimulation epochs (p=0.10, Wilcoxon signed rank test, single units: n=18).

Supplementary Fig. 3e: Correlations of firing rates in the same spatial locations across two control recording sessions compared to control vs. optogenetic stimulation sessions (t_{30}=-0.67, p=0.51, t-test, n=14 single units, control, n=18 single units, optogenetic stimulation, N=4 mice).

Supplementary Fig. 4a: Running speed during epochs of optogenetically entrained hippocampal theta at 7 Hz and spontaneous theta epochs (5-10 Hz). Two-way

repeated measures ANOVA, with factors “experimental subject” and “type of optical stimulation (intra-Hip theta-entraining stimulation vs. control blue light stimulation)”, revealed a significant decrease in running speed during optogenetic theta entrainment, compared to control light stimulation ($F_{1,323}$=18.88, p=0.000019, N=5 mice).

Supplementary Fig. 4c: The duration of continuous running episodes of optogenetically entrained hippocampal theta and spontaneous theta epochs (5-10 Hz). Repeated measures ANOVA, with factors “experimental subject”, “type of optical stimulation (intra-Hip theta-entraining stimulation vs. control blue light stimulation)” and "stimulation frequency", revealed no effect of optogenetic theta entrainment at 7 Hz and 9 Hz on the duration of continuous running epochs ($F_{1,244}$=0.002, p=0.97, N=5 mice) and total time exploring ($F_{1,244}$=0.37, p=0.54).

Supplementary Fig. 4d: Running speed during non-theta frequency optogenetic stimulation of MS hippocampal projections. Repeated measures ANOVA, with factors “experimental subject”, “type of optical stimulation (intra-Hip vs. control blue light stimulation)” and "stimulation frequency", revealed no difference in running speed between types of optical stimulation ($F_{1,131}$=0.31, p=0.58), stimulation frequencies ($F_{2,131}$=0.08, p=0.93) and no interaction between the factors ($F_{2,131}$=1.17, p=0.32; 2 Hz: n=73 recording epochs, N=8 mice; 4 Hz: n=28 recording epochs, N=6 mice; 20 Hz: n=43 recording epochs, N=6 mice). In some of these experiments mice spent more time in the enclosure (>10 min) prior to recordings, resulting in a slightly, not significantly ($F_{3,223}$=0.62, p=0.6) lower average running speed due to accommodation. In the shown in the figure subset of these experiments where average running speed during control recordings was similar to that in Fig. 2c (>5 cm s^{-1}), repeated measures ANOVA, with factors “experimental subject”, “type of optical stimulation (intra-Hip vs. control blue light stimulation)” and "stimulation frequency", also revealed no difference in running speed between types of optical stimulation ($F_{2,84}$=0.07, p=0.8), stimulation frequencies ($F_{2,84}$=2.8, p=0.06) and no interaction between the factors ($F_{2,84}$=1.5, p=0.23; 2 Hz: n=47 recording epochs, N=7 mice; 4 Hz: n=19 recording epochs, N=5 mice; 20 Hz: n=29 recording epochs, N=5 mice).

Supplementary Fig. 4e: Running speed during stimulation with 1 second light pulses and during control light stimulation. Two-way repeated measures ANOVA, with factors “experimental subject” and “type of optical stimulation (intra-Hip vs. control blue light stimulation)”, revealed no effect of continuous 1 sec optogenetic stimulation

on running speed ($F_{1,15}$=1.22, p=0.29, optogenetic stimulation: n=20 recording sessions, N=10 mice; control: n=10 recording sessions, N=9 mice).

Supplementary Fig. 4i: The variability of average running speed upon control light stimulation onset did not differ from the speed variability during randomly selected light-off running epochs (two-sample F-test for equal variances, $F_{41,41}$=0.9, p=0.75, n=42 recording sessions, N=13 mice).

Supplementary Fig. 4j: Running speed variability during non-theta frequency optogenetic stimulation of MS hippocampal projections. Repeated measures ANOVA, with factors "experimental subject", "type of optical stimulation (intra-Hip vs. control blue light stimulation)" and "stimulation frequency", revealed no difference in running speed variability between types of optical stimulation ($F_{1,131}$=0.43, p=0.51), stimulation frequencies ($F_{2,131}$=0.42, p=0.66) and no interaction between the factors ($F_{2,131}$=1.75, p=0.18; 2 Hz: n=73 recording epochs, N=8 mice; 4 Hz: n=28 recording epochs, N=6 mice; 20 Hz: n=43 recording epochs, N=6 mice).

Supplementary Fig. 4k: Running speed variability during stimulation with 1 second light pulses and during control light stimulation. Two-way repeated measures ANOVA, with factors "experimental subject" and "type of optical stimulation (intra-Hip vs. control blue light stimulation)", revealed an increase in running speed variability during optogenetic stimulation with 1 second continuous pulses ($F_{1,15}$=4.67, p=0.047, optogenetic stimulation: n=20 recording sessions, N=10 mice; control: n=10 recording sessions, N=9 mice).

Supplementary Fig. 5c: Spontaneous theta amplitude (5-10 Hz band-pass filtered, amplitude derived from Hilbert transform) in the dorsal but not ventral hippocampus correlated with animal's speed (Pearson's correlation, dHip: r=0.59, p<0.0001, n=6 recording sessions, N=3 mice; vHip: r=-0.12, p=0.45, n=6 recording sessions, N=3 mice).

Supplementary Fig. 6f: Running speed following intra-LS CNO or vehicle administration (running speed >2 cm s-1). Two-way repeated measures ANOVA, with factors "experimental subject" and "drug treatment (intra-LS CNO or vehicle)", revealed that running speed was significantly increased following CNO administration in comparison to vehicle administration ($F_{1,15095}$=143.85, p<0.00001, N=6 mice).

Supplementary Fig. 6i: Running speed variability (CV) during optogenetic hippocampal theta entrainment in the absence of eNpHR3.0 stimulation in the LS. Two-way ANOVA, with factors "experimental subject" (random factor, different mice

in groups) and “yellow light (off vs. intra-LS in eNpHR3.0 (-) mice vs. dummy patch cord in eNpHR3.0 (+) mice)” (fixed factor), revealed no differences in running speed variability ($F_{2,66}$=0.037, p=0.96, N=13 mice).

Supplementary Table 1: Partial Pearson correlations (n=422 recording epochs, N=9 mice).
